# Potential gene identification and pathway crosstalk analysis of age-related macular degeneration

**DOI:** 10.3389/fgene.2022.992328

**Published:** 2022-09-06

**Authors:** Chengda Ren, Jing Yu

**Affiliations:** Department of Ophthalmology, Shanghai Tenth People’s Hospital, School of Medicine, Tongji University, Shanghai, China

**Keywords:** GO analyses, pathway crosstalk, gene identification, AMD, lipid metabolism

## Abstract

Age-related macular degeneration (AMD), the most prevalent visual disorder among the elderly, is confirmed as a multifactorial disease. Studies demonstrated that genetic factors play an essential role in its pathogenesis. Our study aimed to make a relatively comprehensive study about biological functions of AMD related genes and crosstalk of their enriched pathways. 1691 AMD genetic studies were reviewed, GO enrichment and pathway crosstalk analyses were conducted to elucidate the biological features of these genes and to demonstrate the pathways that these genes participate. Moreover, we identified novel AMD-specific genes using shortest path algorithm in the context of human interactome. We retrieved 176 significantly AMD-related genes. GO results showed that the most significant term in each of these three GO categories was: signaling receptor binding (P_BH_ = 4.835 × 10^−7^), response to oxygen-containing compound (P_BH_ = 2.764 × 10^−21^), and extracellular space (P_BH_ = 2.081 × 10^−19^). The pathway enrichment analysis showed that complement pathway is the most enriched. The pathway crosstalk study showed that the pathways could be divided into two main modules. These two modules were connected by cytokine-cytokine receptor interaction pathway. 42 unique genes potentially participating AMD development were obtained. The aberrant expression of the mRNA of FASN and LRP1 were validated in AMD cell and mouse models. Collectively, our study carried out a comprehensive analysis based on genetic association study of AMD and put forward several evidence-based genes for future study of AMD.

## Introduction

Age-related macular degeneration (AMD) is a major cause of irreversible blindness and visual impairment in the elderly of industrialized countries (Gehrs et al., 2006; Klein et al., 2011). AMD leads to progressive central vision loss because of macular atrophy and choroidal neovascularization (Lambert et al., 2016). Currently, no efficient medical or surgical treatment is available for geographic atrophy (GA), also known as the “dry” form of AMD, while anti-vascular endothelial growth factor (VEGF) therapies have been used for treating neovascular AMD, also known as the “wet” form (Campa and Harding, 2011). As one of the most severe eye diseases, the mechanisms of AMD pathogenesis remain elusive.

In the past several decades, researches have demonstrated that AMD is a multi-factorial disease. Both genetic and environmental factors influence the development of AMD. Many risk factors have been confirmed to contribute to AMD progression, including aging, smoking, oxidative stress, sunlight exposure, and genetic factors (Lambert et al., 2016). Identification of risk factors has become one of the main aspects of AMD research in recent years due to their strong correlation with prevalence of AMD. One study showed that the risk of developing late AMD was increased approximately 4-fold for those with a family history of AMD (Smith and Mitchell, 1998). Also, numerous studies about gene polymorphism have been carried out. They have elucidated a lot different genetically susceptive factors for AMD, such as complement factor H (CFH) (Klein et al., 2013), Apolipoprotein E (APOE) (McKay et al., 2011), vascular endothelial growth factor (VEGF) (Miller et al., 2013), and hepatic lipase (LIPC) (Neale et al., 2010). Despite considerable success in deciphering AMD genetic risk factors, the intact mechanism is still veiled. Recently, a meta-analysis of genome-wide association studies (GWAS) for advanced AMD estimated that currently identified loci account for nearly 55% of the heritability of advanced AMD (Yu et al., 2011). On the one hand, a complicated disease tends to be influenced by lots of genes with small or mild effects rather than one or two major genes with large effects. A comprehensive analysis of potentially causal genes within a pathway and/or a network framework might provide some important insights beyond the conventional single-gene analyses (Goeman and Buhlmann, 2007; Glazko and Emmert-Streib, 2009; Jia et al., 2011b; Hu et al., 2017). On the other hand, the disease proteins always tend to interact with each other instead of scattering randomly in the human interactome and form one or several connected subgraphs (Xu and Li, 2006; Goh et al., 2007; Feldman et al., 2008). So, identification of existing AMD-related genes and delineation of the AMD subnetwork may enable us to predict the potential AMD-associated genes, which provide us a more thorough understanding of AMD pathogenesis.

In this study, we firstly established a relatively ample collection of genes genetically associated with AMD. Then, we performed functional enrichment analyses to identify the significant gene ontology (GO) terms and pathways within these retrieved genes. To further explore the pathogenesis of AMD in a more specific manner, we analyzed the crosstalk of AMD-related pathways. Moreover, AMD-associated subnetwork was extracted using shortest path algorithm in the context of the human protein-protein interactome. Subsequently, we made a prediction of candidate genes based on the betweenness in the AMD-specific network. This study provides insights in pathogenesis of AMD and contributes to identify novel genes related with AMD.

## Materials and methods

### Identification of AMD-Related genes

Candidate genes associated with AMD were collected by retrieving the human genetic association studies deposited in PUBMED (http://www.ncbi.nlm.nih.gov/pubmed/). Similar with references (Sullivan et al., 2004; Hu et al., 2017), we searched for studies about AMD with the term (age-related macular degeneration [MeSH]) and (polymorphism [MeSH] or genotype [MeSH] or alleles [MeSH]) not (neoplasms [MeSH]). By 4 January 2020, a total of 1,691 publications were retrieved for the disorder. We reviewed the abstract of all 1,691 publications to select genetic association studies of AMD. Among the selected publications, we only focused on the genes that are statistically significantly related to the incidence of AMD. Moreover, we reviewed the full report of publications that contain significant association to ensure the conclusion was supported by the research. After reviewing, we incorporated those genes into our study and set up a gene collection named AMDgset.

### Functional enrichment analysis of AMD-Related genes

The functional feature of the AMD-related genes were analyzed by ToppGene (Chen et al., 2009). ToppGene is a web-based system that contains information from different resources and is able to be used in detecting the biological themes out of the candidate gene lists, including evaluating the enrichment significance of GO terms. Here, we employed the criterion that only the GO terms of biological processes with both *p* value and false discovery rate (FDR) value smaller than 0.05 were accepted as the significantly enriched GO term. *p* values were calculated with Fisher’s exact test and FDR values were performed by Benjamini and Hochberg (BH) method (P_*BH*_). Due to the advantages of combining multi-databases, ToppGene was also selected to analyze the pathways enriched in the candidate genes. Basically, we uploaded the genes with their symbols and/or corresponding NCBI Entrez Gene IDs into the server and compared with the genes included in each canonical pathway based on the Kyoto Encyclopedia of Genes and Genomes (KEGG; www.genome.jp/kegg) and Biocarta (www.biocarta.com) pathway databases. All the pathways contained two or more candidate genes were extracted, with each of them assigned a *p* value to denote overlap significance between the pathway and the input genes via Fisher’s exact test. Thereafter, we only considered the pathways with FDR value less than 0.05 as significantly enriched pathways. FDR values were also performed by BH method (P_*BH*_).

### Pathway crosstalk analysis

Crosstalk analysis between pathways was evaluated by the Jaccard Coefficient (JC) = $\left|\frac{A\cap B}{A\cup B}\;\right|$ and the Overlap Coefficient (OC) = $\frac{\left|A\cap B\right|}{\min\left(\left|A\right|,\left|B\right|\right)}$ , where A and B is the list of genes included in the two tested pathways. Here we administrate the following procedure to establish the pathway crosstalk:

1. Select a set of pathways for crosstalk analysis. Only the pathways with P_*BH*_ value less than 0.05 were used. Meanwhile, pathways containing less than two candidate genes were removed because pathways with too few genes might have insufficient biological information.  
2. Count the number of shared candidate genes between any pair of pathways. Pathway pair with less than two overlapped genes was removed.  
3. Calculate the overlap of all pathway pairs and rank them. All the pathway pairs were ranked according to their JC and OC value.  
4. Visualize the selected pathway crosstalk with the software Cytoscape [35].  

### Identification of AMD-specific genes based on human interactome

The disease proteins (the products of disease genes) are not dispersed randomly in the interactome, but tend to interact with each other, forming one or several connected subinteractome that we call the disease module. A total of 176 genes were already included in AMD disease module in our study. To identify novel AMD-related genes, we firstly adopted a relatively complete human interactome from a recent study which contained 138,427 physical interactions between 13,460 proteins, including protein-protein and regulatory interactions, metabolic pathway interactions, and kinase-substrate interactions (Menche et al., 2015). Secondly, Subnet, a Java-based stand-alone program for extracting subnetworks using the pairwise K-shortest path algorithms, was employed to extract AMD-specific genes (Lemetre et al., 2013). Here, we used the concept of betweenness (the number of shortest paths connect all pairs of genes in AMDgset and the path should contain a given gene as an inner gene) to evaluate novel AMD associated genes. It is possible that genes with high betweenness may participate more pathological processes of AMD than those with low betweenness. As a gene in a given network, its betweenness may be influenced by the primary structure of the network. For instance, the cut-vertex of the network may always have high betweenness regardless of the distribution of known genes, therefore, a permutation test was conducted to eliminate this phenomenon. We randomly selected the same number of genes as the number of AMDgset from human interactome 100 times and recalculated the shortest paths between these randomly selected genes. The permutation FDR of the shortest path genes was defined as.

FDR_*i*_ = $\frac{\text{count}\;\left({\text{betweenness}}_{\text{random}}>{\text{betweenness}}_{\text{actual}}\right)}{100}$ , where betweenness_actual_ and betweenness_random_ was the number of shortest paths that across gene *i* among AMDgset and randomly selected genes respectively. Count (betweenness_actual_ > betweenness_random_) denoted the count of times when betweenness_random_ was greater than betweenness_actual_. According to Jiang et al.’s work, only genes with betweenness_actual_ > 1,000 and FDR <0.05 were included. Besides, significant AMD specific genes should meet the criteria that count (betweenness_random_) < 50 so that we could furtherly exclude hub genes in the background network (Jiang et al., 2013).

### Cell culture

Adult human RPE cell line ARPE-19 cell was purchased from MEISENCTCC company (Hangzhou, China). DMEM/F12 culture media (Thermo Fisher Scientific) with 10% fetal bovine serum (FBS, Gibco, Carlsbad, CA, United States), 100 U/mL penicillin and 100 mg/ml streptomycin was used in cell culture. All cells were incubated at 37°C under an atmosphere of 5% CO_2_. For further analysis, cells were seeded in 6- or 96-well plate as needed.

### Cell viability assay

After the Sodium iodate (SI, Sigma-aldrich, San Francisco, CA, United States) treatment, the cell viability was measured with CCK-8 kit (Yeasen, Shanghai, China) according to the manufacturer’s protocol then was detected with a microplate reader (BioTek, VT, United States). Propidium Iodide (PI) staining assay was also used to evaluate the cell viability. Briefly, after treatment, cells were incubated with PI (10 μg/ml) and Hoechst for 10 min before imaging at 550 nm.

### Mice

C57BL/6J male mice (6–8 weeks old) were purchased from Beijing Vital River Laboratory Animal Technology (Beijing, China). The animal experiments were all performed according to the ARRIVE guidelines and the ARVO Statement for the Use of Animals in Ophthalmic and Vision. All animal experiments were authorized by the ethical committee of Shanghai 10th People’s Hospital. All animals were given free access to food and drinking water. Mice were housed in a pathogen-free room with constant temperature (22°C) under a 12 h light-dark cycle. SI was dissolved in sterile saline at the concentrations of 4 mg/ml. The solution was given as a single dose at the concentration of 40 mg/kg intraperitoneally. The mice were sacrificed after 2 days.

### Hematoxylin and eosin staining

The mice were sacrificed after 2 days and eyes were fixed in 4% paraformaldehyde for 24 h. After fixation, paraffin-embedded serially sections of 3 μm were cut carefully and then stained with hematoxylin-eosin (H&E). Photos of the sections were taken using an upright light microscope (Leica Microsystems).

### Quantitative PCR

After treatment, total RNA was extracted by EZ-press RNA purification Kit (Roseville, MN, United States) and RNA concentration was determined with NanoDrop 3,300 (Thermo Fisher Scientific). cDNA was synthesized from 1 μg of total RNA using HiScript III first Strand cDNA Synthesis Kit (Vazyme, Nanjing, China). The qPCR analysis was performed using ChamQ universal SYBR qPCR Master Mix (Vazyme). The contents of different mRNA targets in different groups were calculated by ΔΔCt method. Primers were synthesized by Sangon Biotech (Sangon Biotech, Shanghai, China). Primers used in the experiments were as follows: human APOA1 (F: 5′- CCC​TGG​GAT​CGA​GTG​AAG​GA-3′; R: 5′- CTG​GGA​CAC​ATA​GTC​TCT​GCC-3′), human FASN (F: 5′- AAG​GAC​CTG​TCT​AGG​TTT​GAT​GC-3′; R: 5′- TGG​CTT​CAT​AGG​TGA​CTT​CCA-3′), human ABCG5 (F: 5′- TGG​ACC​AGG​CAG​ATC​CTC​AAA-3′; R: 5′- CCG​TTC​ACA​TAC​ACC​TCC​CC-3′), human LRP1 (F: 5′- CTA​TCG​ACG​CCC​CTA​AGA​CTT-3′; R: 5′- CAT​CGC​TGG​GCC​TTA​CTC​T-3′), mouse APOA1 (F: 5′- CTT​GGC​ACG​TAT​GGC​AGC​A-3′; R: 5′- CCA​GAA​GTC​CCG​AGT​CAA​TGG-3′), mouse FASN (F: 5′- GGA​GGT​GGT​GAT​AGC​CGG​TAT-3′; R: 5′- TGG​GTA​ATC​CAT​AGA​GCC​CAG-3′), mouse ABCG5 (F: 5′- AGA​GGG​CCT​CAC​ATC​AAC​AGA-3′; R: 5′- CTG​ACG​CTG​TAG​GAC​ACA​TGC-3′), mouse LRP1 (F: 5′- CCA​CTA​TGG​ATG​CCC​CTA​AAA​C-3′; R: 5′- GCA​ATC​TCT​TTC​ACC​GTC​ACA-3′), human NCK1 (F: 5′- CAA​CAT​GCC​CGC​TTA​TGT​GAA-3′; R: 5′- CAT​GAC​GAT​CAC​CTT​TGT​CCC-3′), human PTPN11 (F: 5′- GAA​CTG​TGC​AGA​TCC​TAC​CTC​T-3′; R: 5′- TCT​GGC​TCT​CTC​GTA​CAA​GAA​A-3′), human PNN (F: 5′- GTC​GCC​GTG​AGA​ACT​TTG​C-3′; R: 5′- GGT​CCT​CCT​CCA​CTA​TCT​GAG​A-3′), human CNGB1 (F: 5′- GGA​CCC​CTC​GGA​AGA​CCA​A-3′; R: 5′- CTC​AGG​ATT​CGG​TTC​TGG​TTC-3′).

### Statistical analysis

Each experiment was repeated at least thrice. Graphpad Prism 9 was used to perform statistical analyses. All data was expressed as the mean ± SEM, statistical differences were determined by Student’s t-test for comparison between two groups. *p* < 0.05 was considered to be statistically significant.

## Results

### Retrieve of genes reported to Be associated with AMD

With the criteria described above, publications showing significant association of gene(s) with the disease were collected; those insignificant results were excluded. A detailed list of genes that have been reported to be significantly associated with AMD is provided in Table 1. We constructed a gene set (referred to as AMD-related genes gene set (AMDgset)) which contains 176 genes significantly associated with AMD. Among them, the complement family (C2, C3, C9, CFH, CFHR1, CFHR2) contained the maximum members and was considered to play a pivotal role in AMD pathogenesis. AMDgset also contained cytochrome proteins (CYP1A2, CYP46A1, CYP2R1), vascular endothelial growth factor A (VEGFA), and anti-oxidative proteins (SOD2, SOD3), which are highly associated with intraretinal environment. At the meantime, some other proteins such as collagen family (COL4A3, COL8A1, COL10A1, COL15A1), matrix metallopeptidase (MMP2, MMP9, MMP20), and toll like receptor (TLR2, TLR3, TLR4) were also reported to be associated with AMD. Our results showed the diversity of AMD related genes and indicated the multifactorial characteristic of AMD in terms of genetics.

**TABLE 1:** Genes retrieved from human genetic association studies.

Gene Symbol	Gene ID	Full Name
ABCG1	9619	ATP binding cassette subfamily G member 1
ABCG8	64241	ATP binding cassette subfamily G member 8
ABHD2	11057	abhydrolase domain containing 2
ACAD10	80724	acyl-CoA dehydrogenase family member 10
ACE	1636	angiotensin I converting enzyme
ADAMTS9	56999	ADAM metallopeptidase with thrombospondin type 1 motif 9
ALDH3A2	224	aldehyde dehydrogenase 3 family member A2
ANGPT2	285	angiopoietin 2
APOE	348	apolipoprotein E
ARHGAP21	57584	Rho GTPase activating protein 21
ARMS2	387715	age-related maculopathy susceptibility 2
ASPM	259266	abnormal spindle microtubule assembly
B3GLCT	145173	beta 3-glucosyltransferase
BCO1	53630	beta-carotene oxygenase 1
BCO2	83875	beta-carotene oxygenase 2
C2	717	complement C2
C20orf85	128602	chromosome 20 open reading frame 85
C3	718	complement C3
C4A	720	complement C4A (Rodgers blood group)
C6orf223	221416	chromosome 6 open reading frame 223
C9	735	complement C9
CACNG3	10368	calcium voltage-gated channel auxiliary subunit gamma 3
CAPN5	726	calpain 5
CATSPER2	117155	cation channel sperm associated 2
CCL2	6347	C-C motif chemokine ligand 2
CCR2	729230	C-C motif chemokine receptor 2
CCR3	1232	C-C motif chemokine receptor 3
CD36	948	CD36 molecule
CD63	967	CD63 molecule
CETP	1071	cholesteryl ester transfer protein
CFB	629	complement factor B
CFD	1675	complement factor D
CFH	3075	complement factor H
CFHR1	3078	complement factor H related 1
CFHR2	3080	complement factor H related 2
CFHR3	10878	complement factor H related 3
CFHR4	10877	complement factor H related 4
CFHR5	81494	complement factor H related 5
CFI	3426	complement factor I
CLUL1	27098	clusterin like 1
CNN2	1256	calponin 2
COL10A1	1300	collagen type X alpha 1 chain
COL15A1	1306	collagen type XV alpha 1 chain
COL4A3	1285	collagen type IV alpha 3 chain
COL8A1	1295	collagen type VIII alpha 1 chain
CRP	1401	C-reactive protein [*Homo sapiens*
CST3	1471	cystatin C
CTRB1	1504	chymotrypsinogen B1
CTRB2	440387	chymotrypsinogen B2
CX3CR1	13051	chemokine (C-X3-C motif) receptor 1
CXCL8	3576	C-X-C motif chemokine ligand 8
CYP1A2	1544	cytochrome P450 family 1 subfamily A member 2
CYP2R1	120227	cytochrome P450 family 2 subfamily R member 1
CYP46A1	10858	cytochrome P450 family 46 subfamily A member 1
DAPL1	92196	death associated protein like 1
DDR1	780	discoidin domain receptor tyrosine kinase 1
ELN	2006	elastin
ELOVL4	6785	ELOVL fatty acid elongase 4
ERCC2	2068	ERCC excision repair 2, TFIIH core complex helicase subunit
ERCC6	2074	ERCC excision repair 6, chromatin remodeling factor
ESR1	2099	estrogen receptor 1
F13B	2165	coagulation factor XIII B chain
FADS1	3992	fatty acid desaturase 1
FADS2	9415	fatty acid desaturase 2
FBLN5	10516	fibulin 5
FCGR2A	2212	Fc fragment of IgG receptor IIa
FGD6	55785	FYVE, RhoGEF and PH domain containing 6
FGL1	2267	fibrinogen like 1
FILIP1L	11259	filamin A interacting protein 1 like
FKBPL	63943	FK506 binding protein like
FLT1	2321	fms related tyrosine kinase 1
FPR1	2357	formyl peptide receptor 1
FRK	2444	fyn related Src family tyrosine kinase
GAS6	2621	growth arrest specific 6
GPX1	2876	glutathione peroxidase 1
GPX3	2878	glutathione peroxidase 3
GRK5	2869	G protein-coupled receptor kinase 5
GSTM1	2944	glutathione S-transferase mu 1
HLA-B	3106	major histocompatibility complex, class I, B
HLA-C	3017	major histocompatibility complex, class I, C
HLA-DQB1	3119	major histocompatibility complex, class II, DQ beta 1
HMCN1	83872	hemicentin 1
HMOX1	3162	heme oxygenase 1
HMOX2	3163	heme oxygenase 2
HTRA1	5654	HtrA serine peptidase 1
IER3	8870	immediate early response 3
IGF1R	3480	insulin like growth factor 1 receptor
IL17A	3605	interleukin 17A
IL17RC	84818	interleukin 17 receptor C
IL1B	3553	interleukin 1 beta
KCTD10	83892	potassium channel tetramerization domain containing 10
KDR	3791	kinase insert domain receptor
KMT2E	55904	lysine methyltransferase 2E
LIPC	3990	lipase C, hepatic type
LOXL1	4016	lysyl oxidase like 1
LRP6	4040	LDL receptor related protein 6
MALL	7851	mal, T cell differentiation protein like
MMP2	4313	matrix metallopeptidase 2
MMP20	9313	matrix metallopeptidase 20
MMP9	4318	matrix metallopeptidase 9
MRPL10	124995	mitochondrial ribosomal protein L10
MT2A	4502	metallothionein 2A
MTHFR	4524	methylenetetrahydrofolate reductase
MTR	4548	5-methyltetrahydrofolate-homocysteine methyltransferase
MYRIP	25924	myosin VIIA and Rab interacting protein
NFE2L2	4780	nuclear factor, erythroid 2 like 2
NOS2	4843	nitric oxide synthase 2
NOS3	4846	nitric oxide synthase 3
NPC1L1	29881	NPC1 like intracellular cholesterol transporter 1
NPHP1	4867	nephrocystin 1
NPLOC4	55666	NPL4 homolog, ubiquitin recognition factor
NQO1	1728	NAD(P)H quinone dehydrogenase 1
OSBP2	23762	oxysterol binding protein 2
P2RX4	5025	purinergic receptor P2X 4
P2RX7	5027	purinergic receptor P2X 7
PGF	5228	placental growth factor
PILRA	29992	paired immunoglobin like type 2 receptor alpha
PILRB	29990	paired immunoglobin like type 2 receptor beta
PLEKHA1	59338	pleckstrin homology domain containing A1
PON1	5444	paraoxonase 1
PPARG	5468	peroxisome proliferator activated receptor gamma
PPARGC1A	10891	PPARG coactivator 1 alpha
PRKDC	5591	protein kinase, DNA-activated, catalytic polypeptide
PRKN	5071	parkin RBR E3 ubiquitin protein ligase
PRLR	5618	prolactin receptor
PTCHD3	374308	patched domain containing 3
RAD51	5888	RAD51recombinase
RAD51B	5890	RAD51 paralog B
RDH5	5959	retinol dehydrogenase 5
RGS10	6001	regulator of G protein signaling 10
RHO	6010	rhodopsin [Homo sapiens
RLBP1	6017	retinaldehyde binding protein 1
ROBO1	6091	roundabout guidance receptor 1
RORA	6095	RAR related orphan receptor A
RORB	6096	RAR related orphan receptor B
RXRA	6256	retinoid X receptor alpha
SCARB1	949	scavenger receptor class B member 1
SELP	6403	selectin P
SERPINF1	5176	serpin family F member 1
SERPING1	710	serpin family G member 1
SIRT1	23411	sirtuin 1
SKIV2L	6499	Ski2 like RNA helicase
SLC16A8	23539	solute carrier family 16 member 8
SLC44A4	80736	solute carrier family 44 member 4
SMUG1	23583	single-strand-selective monofunctional uracil-DNA glycosylase
SOD2	6648	superoxide dismutase 2
SOD3	6649	superoxide dismutase 3
SPEF2	79925	sperm flagellar 2
SRPK2	6733	SRSF protein kinase 2
STRC	161497	stereocilin
SYN3	8224	synapsin III
TF	7018	transferrin
TFR2	7036	transferrin receptor 2
TFRC	7037	transferrin receptor
TGFBR1	7046	transforming growth factor beta receptor 1
TIMP3	7078	TIMP metallopeptidase inhibitor 3
TLR2	7097	toll like receptor 2
TLR3	7098	toll like receptor 3
TLR4	7099	toll like receptor 4
TMEM97	27346	transmembrane protein 97
TNF	7124	tumor necrosis factor
TNFRSF10A	8797	TNF receptor superfamily member 10a
TNMD	64102	tenomodulin
TNXB	7148	tenascin XB
TRPM1	4308	transient receptor potential cation channel subfamily M member 1
TRPM3	80036	transient receptor potential cation channel subfamily M member 3
TSPAN10	83882	tetraspanin 10
UBE3D	90025	ubiquitin protein ligase E3D
UNG	7374	uracil DNA glycosylase
VDR	7421	vitamin D receptor
VEGFA	7422	vascular endothelial growth factor A
VLDLR	7436	very low density lipoprotein receptor
VTN	7448	vitronectin
ZBTB41	226470	zinc finger and BTB domain containing 41

### Gene ontology enrichment analysis

**FIGURE 1:**
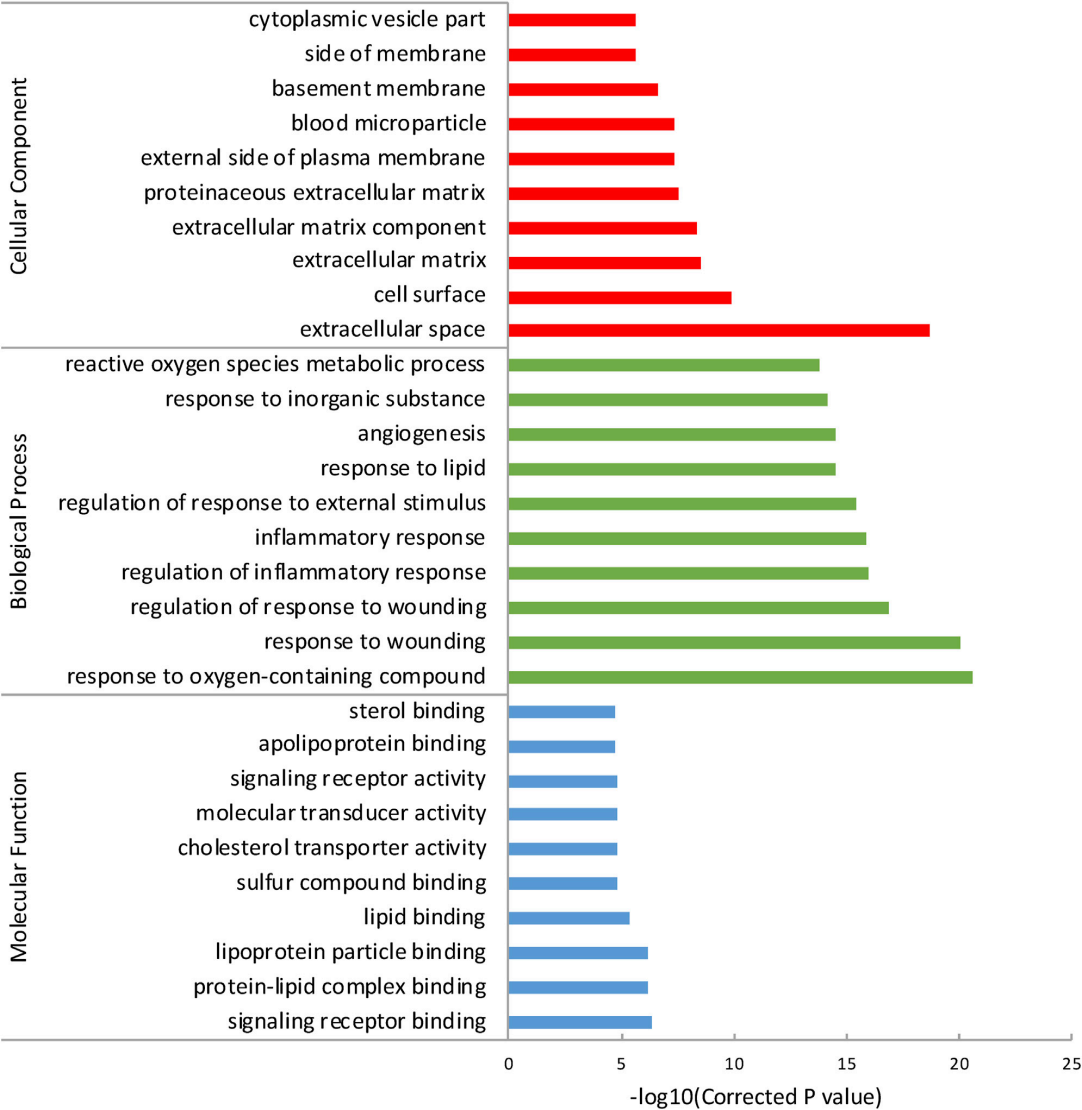
The top 10 GO terms of each category. The GO terms were divided into 3 parts according to cellular component, biological process and molecular function.

To reveal a more specifically functional feature of these genes, we performed GO enrichment analysis with ToppGene and incorporated the top 10 GO terms of each category (Table 2). Results showed that the most significant term in each of these three GO categories was: signaling receptor binding (P_BH_ = 4.835 × 10^−7^), response to oxygen-containing compound (P_BH_ = 2.764 × 10^−21^), and extracellular space (P_BH_ = 2.081 × 10^−19^), respectively (Figure 1). It has long been presumed that aberration of cytokine-cytokine receptor activation is the main early AMD manifestation as mononuclear phagocytes (MPs) are observed on large drusen (Combadiere et al., 2007). Moreover, immunostaining of central retinal pigment epithelium (RPE) flatmounts reveal that IBA-1^+^ MPs and CCR2^+^ monocytes (Mos), can be detected within geographic zone and on drusen, are seldom present in healthy age-matched central donor RPE (Sennlaub et al., 2013; Eandi et al., 2016). These atypical appearances of monocytes can be explained by a combination of abnormal signaling receptor binding, including age-related increase of CCL2, deficiency of CX3CL1 as well as pro-inflammatory pattern of interleukins (Guillonneau et al., 2017). We also noticed that lipid (e.g., protein-lipid complex binding, lipoprotein particle binding, lipid binding), oxidative (e.g., response to oxygen-containing compound, reactive oxygen species metabolic process) and extracellular matrix (ECM) (e.g., ECM, ECM component, proteinaceous ECM) related GO terms were enriched in the genes of AMDgset. These results were in accordance with previous researches which demonstrated lipid deposition, oxidative stress, and ECM alteration played prominent roles in AMD pathogenesis (Nita et al., 2014; Jun et al., 2019). Our GO results indicated the AMDgset is relatively reliable for subsequent analysis.

**TABLE 2:** Gene Ontology (GO) terms enriched with AMDgset (Top 10 terms).

Go terms	*P* ^a^	*P*_BH_^b^	Observed
*Molecular Function*			
GO:0005102: signaling receptor binding	5.783×10^-10^	4.835×10^-7^	41
GO:0071814: protein-lipid complex binding	2.408×10^-9^	5.919×10^-8^	7
GO:0071813: lipoprotein particle binding	2.408×10^-9^	6.711×10^-7^	7
GO:0008289: lipid binding	1.89×10^-8^	6.711×10^-7^	24
GO:1901681: sulfur compound binding	1.019×10^-7^	3.949×10^-6^	14
GO:0017127: cholesterol transporter activity	1.045×10^-7^	1.455×10^-5^	5
GO:0060089: molecular transducer activity	1.247×10^-7^	1.455×10^-5^	38
GO:0038023: signaling receptor activity	1.571×10^-7^	1.49×10^-5^	34
GO:0034185: apolipoprotein binding	2.246×10^-7^	1.642×10^-5^	5
GO:0032934: sterol binding	2.282×10^-7^	1.823×10^-5^	7
*Biological Process*			
GO:1901700: response to oxygen-containing compound	5.695×10^-25^	2.764×10^-21^	64
GO:0009611: response to wounding	3.818×10^-24^	9.267×10^-21^	50
GO:1903034: regulation of response to wounding	8.509×10^-21^	1.377×10^-17^	34
GO:0050727: regulation of inflammatory response	8.855×10^-20^	1.075×10^-16^	29
GO:0006954: inflammatory response	1.297×10^-19^	1.259×10^-16^	39
GO:0032101: regulation of response to external stimulus	5.007×10^-19^	4.051×10^-16^	45
GO:0033993: response to lipid	4.416×10^-18^	2.824×10^-15^	44
GO:0001525: angiogenesis	4.654×10^-18^	2.824×10^-15^	31
GO:0010035: response to inorganic substance	1.413×10^-17^	7.622×10^-15^	33
GO:0072593: reactive oxygen species metabolic process	3.054×10^-17^	1.482×10^-14^	24
*Cellular Component*			
GO:0005615: extracellular space	5.038×10^-22^	2.081×10^-19^	57
GO:0009986: cell surface	6.597×10^-13^	1.362×10^-10^	34
GO:0031012: extracellular matrix	2.024×10^-11^	2.786×10^-9^	23
GO:0044420: extracellular matrix component	4.704×10^-11^	4.857×10^-9^	14
GO:0005578: proteinaceous extracellular matrix	3.289×10^-10^	2.717×10^-8^	20
GO:0009897: external side of plasma membrane	5.808×10^-10^	3.998×10^-8^	18
GO:0072562: blood microparticle	7.148×10^-10^	4.217×10^-8^	13
GO:0005604: basement membrane	5.02×10^-9^	2.592×10^-7^	11
GO:0098552: side of membrane	5.07×10^-8^	2.327×10^-6^	20
GO:0044433: cytoplasmic vesicle part	5.038×10^-22^	2.444×10^-6^	22

### Pathway enrichment analysis in AMDgset

**FIGURE 2:**
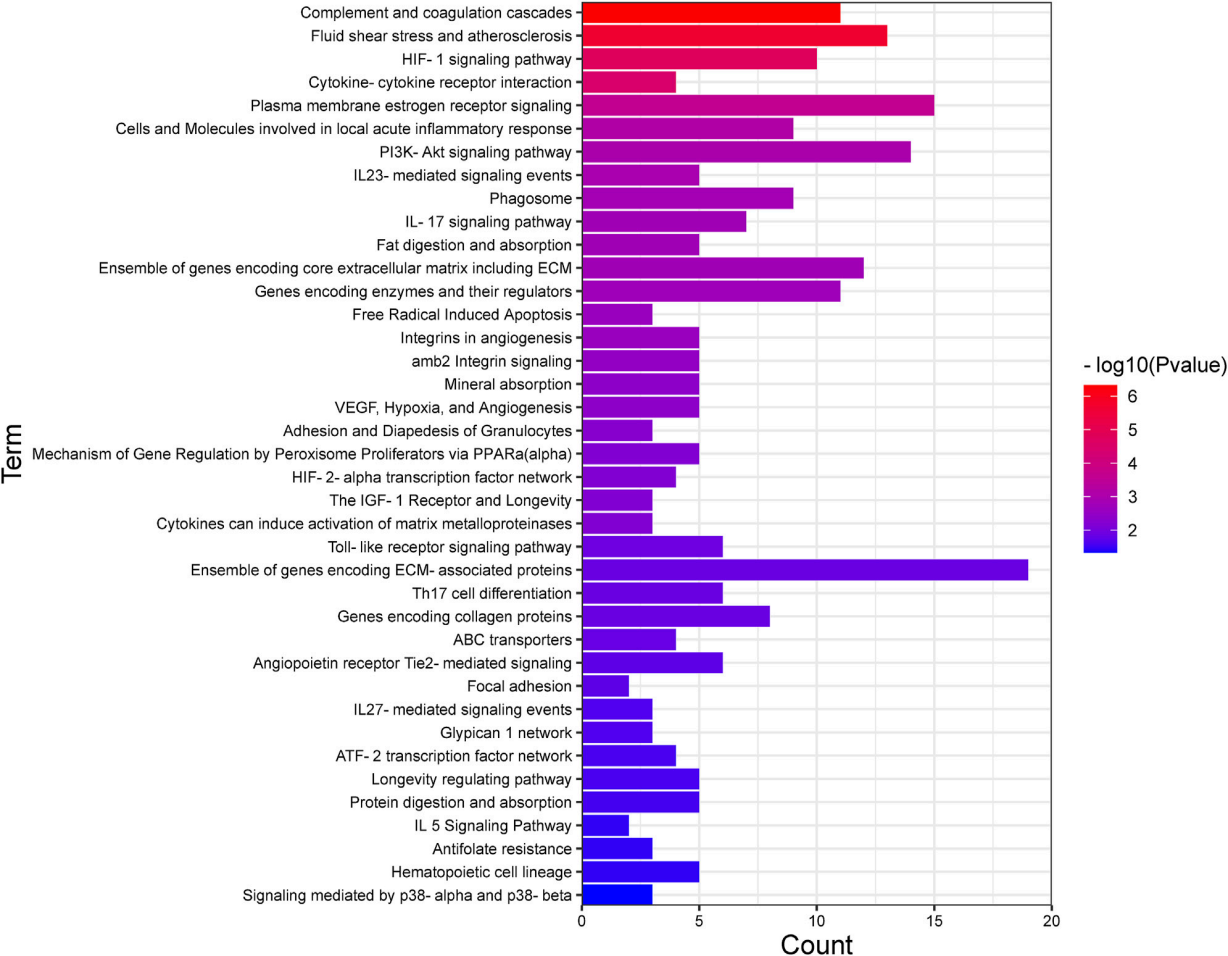
All the pathways enriched in AMDgset ranked by significance.

**FIGURE 3:**
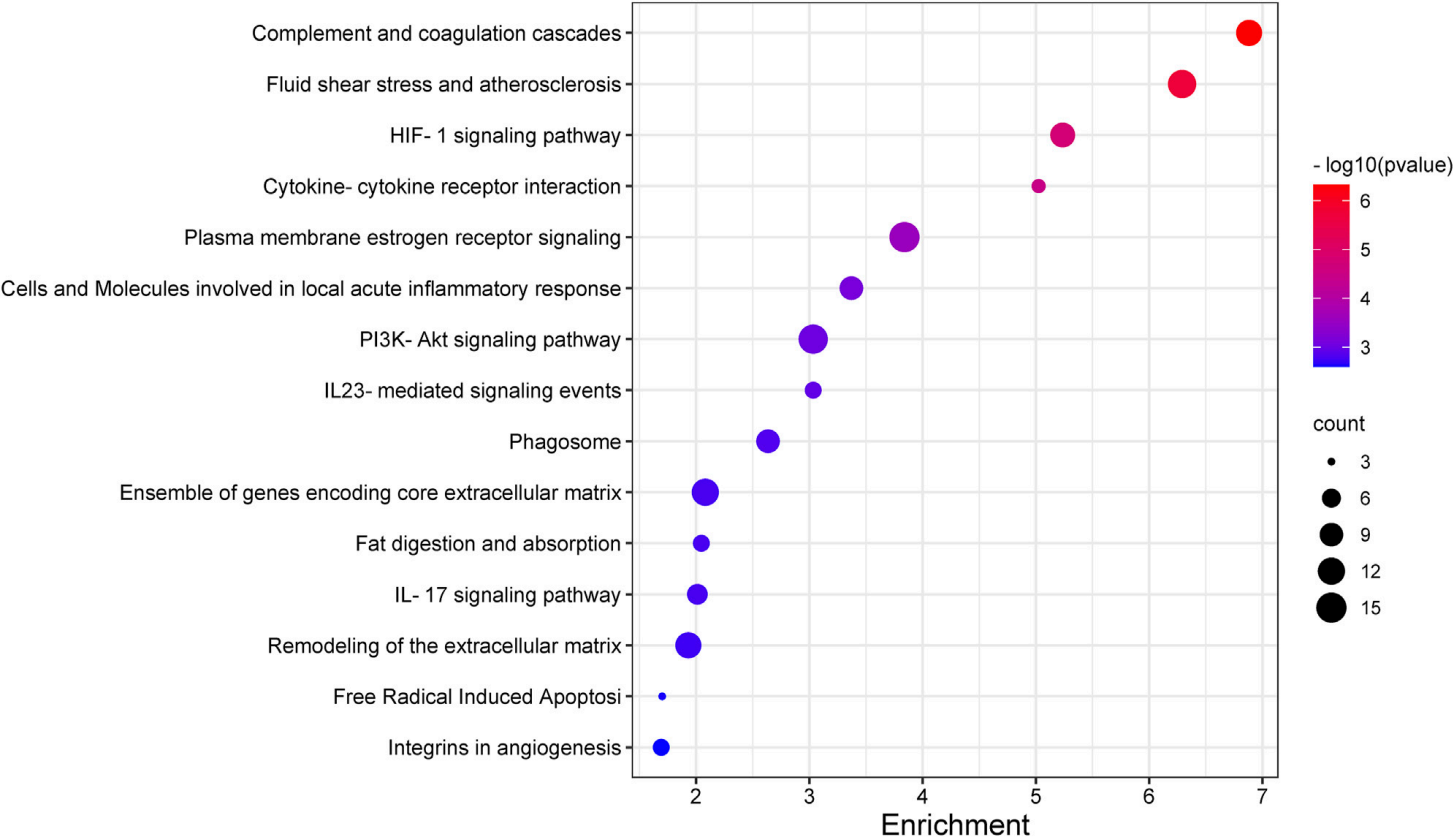
The top 15 pathways enriched in AMDgset.

Recognizing the biochemical pathways enriched in the candidate genes will help us to make a better understanding about the specific intracellular signaling related to AMD. We used ToppGene and found 39 significant enrichment pathways for AMD (Figure 2; Table 3). The top 15 pathways were showed in Figure 3. Since numerous complement related genes were included in AMDgset, complement and coagulation cascades pathway was the most significantly enriched pathway in AMDgset. The result suggested the importance of complement system in the pathogenesis of AMD (Despriet et al., 2009; Baas et al., 2010). Also, results showed that IL-23, IL-17, IL-27 and IL-5 mediated signaling pathways were significantly enriched. IL-17 was confirmed to be elevated in the serum of AMD patients. Coughlin et al. demonstrated that IL-17 could mediate the local inflammation augmenting which is triggered by choroidal neovascularization (CNV) lesions (Coughlin et al., 2016). Moreover, consist with GO analysis, the Fat digestion related pathway was testified as enriched pathway, indicating a prominent role of lipid metabolism in the development of AMD. Furthermore, several canonical pathways such as Free Radical Induced Apoptosis pathway (Jarrett and Boulton, 2012) and VEGF, Hypoxia, and Angiogenesis pathway (Bressler, 2009) were verified in our study as well.

**TABLE 3:** Pathways enriched in AMDgset.

Pathways	*P* ^a^	*P*_BH_^b^	Genes included in Pathways
Complement and coagulation cascades	2.404×10^–9^	4.712×10^–7^	CFH, VTN, CFI, F13B, CFB, CFD, SERPING1, C2, C3, C4A, C9
Fluid shear stress and atherosclerosis	1.532×10^–8^	2.002×10^–6^	HMOX1, HMOX2, GSTM1, NFE2L2, NQO1, CCL2, KDR, TNF, MMP2, MMP9, IL1B, NOS3, VEGFA
HIF–1 signaling pathway	3.59×10^–7^	1.716×10^–5^	FLT1, ANGPT2, HMOX1, TF, TFRC, IGF1R, TLR4, NOS2, NOS3, VEGFA
Cytokine–cytokine receptor interaction	8.867×10^–7^	3.476×10^–5^	FLT1, IL17A, IL17RC, TNFRSF10A, CCR2, TGFBR1, CCL2, KDR, CCR3, TNF, IL1B, PRLR, CX3CR1, CXCL8, VEGFA
Plasma membrane estrogen receptor signaling	1.274×10^–5^	2.628×10^–4^	ESR1, IGF1R, MMP2, MMP9, NOS3
Cells and Molecules involved in local acute inflammatory response	4.264×10^–5^	7.268×10^–4^	SELP, C3, TNF, CXCL8
PI3K–Akt signaling pathway	6.555×10^–5^	1.028×10^–3^	COL4A3, FLT1, VTN, ANGPT2, PGF, RXRA, IGF1R, TLR2, TLR4, KDR, TNXB, NOS3, PRLR, VEGFA
IL23–mediated signaling events	7.585×10^–5^	1.144×10^–3^	IL17A, CCL2, TNF, IL1B, NOS2
Phagosome	1.009×10^–4^	1.465×10^–3^	HLA–B, HLA–DQB1, TFRC, FCGR2A, CD36, SCARB1, TLR2, TLR4, C3
Ensemble of genes encoding core extracellular matrix including ECM glycoproteins, collagens and proteoglycans	1.221×10^–4^	1.668×10^–3^	COL4A3, COL8A1, COL10A1, FBLN5, VTN, COL15A1, GAS6, KERA, HMCN1, ELN, FGL1, TNXB
Fat digestion and absorption	1.254×10^–4^	1.668×10^–3^	ABCA1, CD36, SCARB1, NPC1L1, ABCG8
IL–17 signaling pathway	1.277×10^–4^	1.668×10^–3^	IL17A, IL17RC, CCL2, TNF, MMP9, IL1B, CXCL8
Genes encoding enzymes and their regulators involved in the remodeling of the extracellular matrix	1.426×10^–4^	1.803×10^–3^	HTRA1, SERPINF1, MMP20, F13B, TIMP3, ADAMTS9, LOXL1, CST3, SERPING1, MMP2, MMP9
Free Radical Induced Apoptosis	1.933×10^–4^	2.368×10^–3^	GPX1, TNF, CXCL8
Integrins in angiogenesis amb2 Integrin signaling	2.186×10^–4^	2.521×10^–3^	COL4A3, VTN, IGF1R, KDR, VEGFA
Mineral absorption	2.679×10^–4^	3×10^–3^	SELP, VTN, TNF, MMP2, MMP9
VEGF, Hypoxia, and Angiogenesis	3.57×10^–4^	3.782×10^–3^	HMOX1, HMOX2, TF, MT2A, VDR
Adhesion and Diapedesis of Granulocytes	3.803×10^–4^	3.822×10^–3^	FLT1, KDR, NOS3, VEGFA
Mechanism of Gene Regulation by Peroxisome Proliferators via PPARa(alpha)	5.66×10^–4^	5.283×10^–3^	SELP, TNF, CXCL8
Cytokines can induce activation of matrix metalloproteinases, which degrade extracellular matrix	6.509×10^–4^	5.934×10^–3^	RXRA, PPARGC1A, CD36, TNF, NOS2
The IGF–1 Receptor and Longevity	7.013×10^–4^	6.039×10^–3^	ACE, TNF, IL1B
HIF–2–alpha transcription factor network	7.013×10^–4^	6.039×10^–3^	IGF1R, SOD2, SOD3
Toll–like receptor signaling pathway	7.087×10^–4^	6.039×10^–3^	FLT1, SIRT1, KDR, VEGFA
Ensemble of genes encoding ECM–associated proteins including ECM–affilaited proteins, ECM regulators and secreted factors	1.749×10^–3^	1.224×10^–2^	TLR2, TLR3, TLR4, TNF, IL1B, CXCL8

Pathways	*P* ^a^	*P*_BH_^b^	Genes included in Pathways
Th17 cell differentiation	1.842×10^–3^	1.313×10^–2^	IL17A, HLA–DQB1, RXRA, TGFBR1, RORA, IL1B
Genes encoding collagen proteins	1.89×10^–3^	1.323×10^–2^	COL4A3, COL8A1, COL10A1, COL15A1
ABC transporters	2.055×10^–3^	1.389×10^–2^	ABCA1, ABCA4, ABCG1, ABCG8
Angiopoietin receptor Tie2–mediated signaling	2.61×10^–3^	1.734×10^–2^	ANGPT2, TNF, MMP2, NOS3
Focal adhesion	2.815×10^–3^	1.839×10^–2^	COL4A3, FLT1, VTN, PGF, IGF1R, KDR, TNXB, VEGFA
Glypican 1 network	3.637×10^–3^	2.263×10^–2^	FLT1, TGFBR1, VEGFA
IL27–mediated signaling events	3.637×10^–3^	2.263×10^–2^	IL17A, TNF, IL1B
ATF–2 transcription factor network	4.287×10^–3^	2.626×10^–2^	PPARGC1A, MMP2, NOS2, CXCL8
Longevity regulating pathway	4.378×10^–3^	2.64×10^–2^	PPARG, SIRT1, PPARGC1A, IGF1R, SOD2
Protein digestion and absorption	4.592×10^–3^	2.727×10^–2^	COL4A3, COL10A1, COL15A1, ELN, CTRB1
Antifolate resistance	6.018×10^–3^	3.511×10^–2^	MTHFR, TNF, IL1B
IL 5 Signaling Pathway	6.09×10^–3^	3.511×10^–2^	CCR3, IL1B
Hematopoietic cell lineage	6.298×10^–3^	3.578×10^–2^	HLA–DQB1, TFRC, CD36, TNF, IL1B
Signaling mediated by p38–alpha and p38–beta	8.462×10^–3^	4.672×10^–2^	ESR1, PPARGC1A, NOS2

### Crosstalk among significantly enriched pathways

**FIGURE 4:**
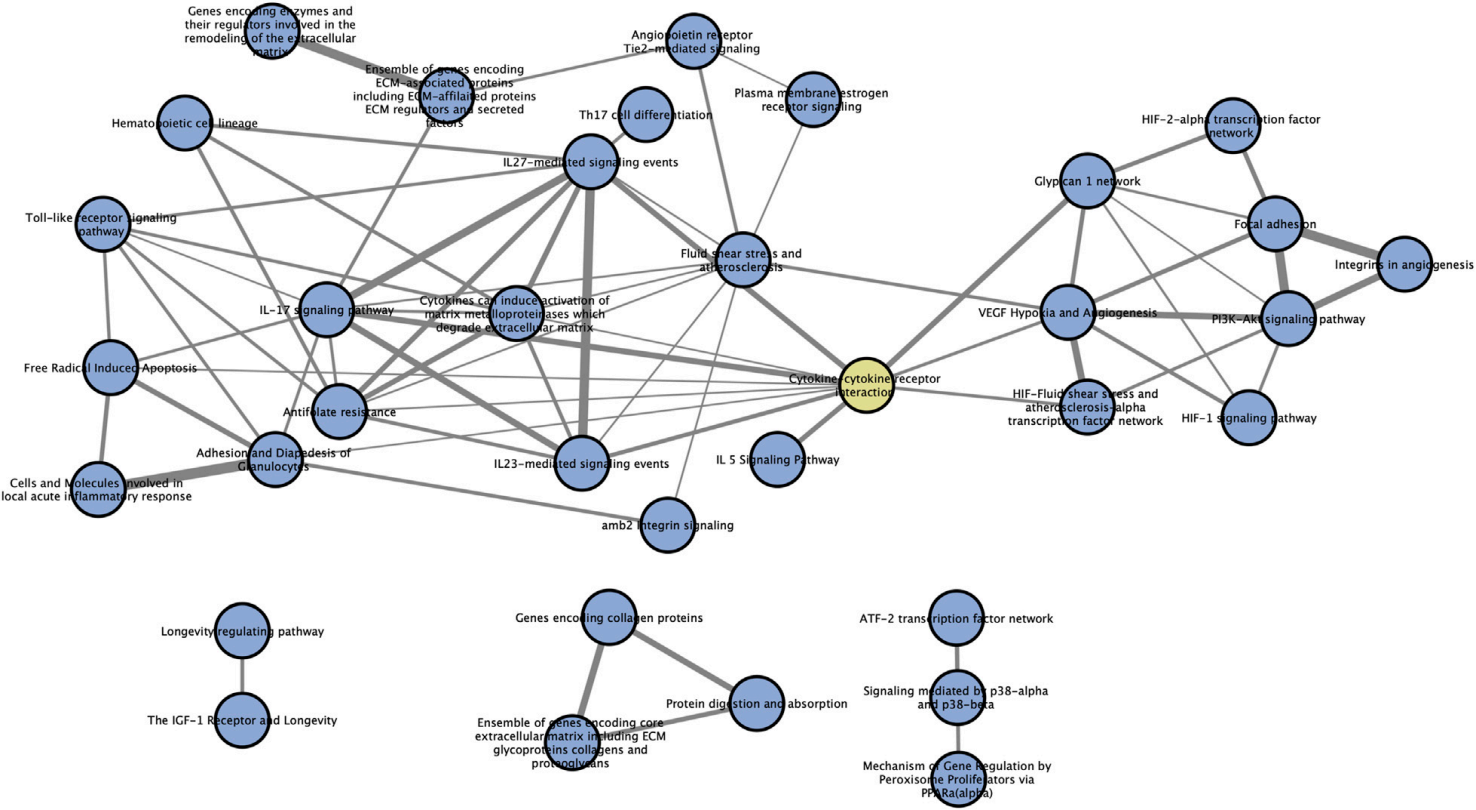
Pathway crosstalk among AMDgset-enriched pathways. Nodes denote pathways while edges represent crosstalk between pathways. The yellow node represents “cytokine-cytokine receptor interaction” pathway which acts as the joint of two main modules. The width of edges is depended on the score of specific pathway pair, wider edge indicates stronger correlation.

Pathways always exert their functions interactively instead of independently. So, we performed a pathway crosstalk analysis among 39 significantly enriched pathways to elaborate their relationships in this disorder. According to the assumption that two pathways were considered to crosstalk if they shared two or more genes of AMDgset (Jia et al., 2011a), we extracted 142 pathway interactions which met the criterion for crosstalk analysis (Table 4). Then we calculated their overlapping level according to the average score of coefficients JC and OC. Furthermore, to make a brief view of the complicate network of pathway crosstalk, we only chose the top 50% overlapped interactions (edges) and their related pathways (nodes) to build the pathway crosstalk (Figure 4). As it was reflected in our results, the pathways could be grouped into two major modules. Each module contained a relatively centralized crosstalk. This phenomenon indicated that the pathways in the same module might take part in a common biological process. The smaller one mainly contained pathways associated with hypoxia, antioxidation and angiogenesis. The bigger module was consisted of pathways related to immune system, inflammation response and ECM. Moreover, results also clearly showed that the two modules were jointed by cytokine-cytokine receptor interaction pathway instead of operating independently.

**TABLE 4:** Pathway crosstalk information.

Pathway A	Pathway B	Score
Cells and Molecules involved in local acute inflammatory response	Adhesion and Diapedesis of Granulocytes Focal adhesion	0.87500
lntegrins in angiogenesis	Focal adhesion	0.81250
Genes encoding enzymes and their regulators involved in the remodeling of the extracellular matrix	Ensemble of genes encoding ECM-associated proteins including ECM-affilaited proteins ECM regulators and secreted factors	0.80556
IL23-mediated signaling events	IL27-mediated signaling events	0.80000
PI3K-Akt signaling pathway	Focal adhesion	0.78571
IL-17 signaling pathway	IL27-mediated signaling events	0.71429
PI3K-Akt signaling pathway	lntegrins in angiogenesis	0.67857
VEGF Hypoxia and Angiogenesis	HIF-Fluid shear stress and atherosclerosis-alpha transcription factor network	0.67500
Ensemble of genes encoding core extracellular matrix including ECM glycoproteins collagens and proteoglycans	Genes encoding collagen proteins	0.66667
IL23-mediated signaling events		
PI3K-Akt signaling pathway	IL-17 signaling pathway	0.65000
Genes encoding collagen proteins	VEGF Hypoxia and Angiogenesis	0.64286
Cytokine-cytokine receptor interaction	Protein digestion and absorption	0.62500
Cytokine-cytokine receptor interaction	IL-17 signaling pathway	0.61607
Cytokine-cytokine receptor interaction	IL27-mediated signaling events	0.60000
Free Radical Induced Apoptosis	Glypican 1 network	0.60000
Cytokines can induce activation of matrix metalloproteinases which degrade extracellular matrix	Adhesion and Diapedesis of Granulocytes	0.58333
Cytokines can induce activation of matrix metalloproteinases which degrade extracellular matrix	IL27-mediated signaling events	0.58333
IL27-mediated signaling events	Antifolate resistance	0.58333
Cytokine-cytokine receptor interaction		
Ensemble of genes encoding core extracellular matrix including ECM glycoproteins collagens and proteoglycans	Antifolate resistance	0.58333
VEGF Hypoxia and Angiogenesis	IL 5 Signaling Pathway	0.56667
HIF-Fluid shear stress and atherosclerosis alpha transcription factor network	Protein digestion and absorption	0.55385
Cells and Molecules involved in local acute inflammatory response	Focal adhesion	0.54167
VEGF Hypoxia and Angiogenesis	Focal adhesion	0.54167
HIF-Fluid shear stress and atherosclerosis alpha transcription factor network	Free Radical Induced Apoptosis	0.53333
ATF-Fluid shear stress and atherosclerosis transcription factor network	Glypican 1 network	0.53333
Cytokine-cytokine receptor interaction	Glypican 1 network	0.53333
HIF-1 signaling pathway	Signaling mediated by p38-alpha and p38-beta	0.53333
IL23-mediated signaling events	IL23-mediated signaling events	0.52500
IL23-mediated signaling events	VEGF Hypoxia and Angiogenesis	0.51136
amb2 Integrin signaling	Cytokines can induce activation of matrix metalloproteinases which degrade extracellular matrix	0.50000
Mechanism of Gene Regulation by Peroxisome Proliferators via PPARa(alpha)		
The IGF-1 Receptor and Longevity	Antifolate resistance	0.50000
	Adhesion and Diapedesis of Granulocytes	0.50000
Cytokines can induce activation of matrix metalloproteinases which degrade extracellular matrix	Signaling mediated by p38-alpha and p38-beta	0.50000
IL27-mediated signaling events	Longevity regulating pathway	0.50000
Antifolate resistance	Hematopoietic cell lineage	0.50000
Fluid shear stress and atherosclerosis		
Fluid shear stress and atherosclerosis	Hematopoietic cell lineage	0.50000
IL-17 signaling pathway	Hematopoietic cell lineage	0.50000
	VEGF Hypoxia and Angiogenesis	0.48214
Free Radical Induced Apoptosis	Angiopoietin receptor Tie2-mediated signaling	0.48214
Adhesion and Diapedesis of Granulocytes	Ensemble of genes encoding ECM-associated proteins including ECM-affilaited proteins ECM regulators and secreted factors	0.48214
Cytokines can induce activation of matrix metalloproteinases which degrade extracellular matrix	Toll-like receptor signaling pathway	0.47619
Toll-like receptor signaling pathway	Toll-like receptor signaling pathway	0.47619
Toll-like receptor signaling pathway	Toll-like receptor signaling pathway	
Th17 cell differentiation		0.47619
PI3K-Akt signaling pathway	IL27-mediated signaling events	0.47619
Cytokine-cytokine receptor interaction	Antifolate resistance	0.47619
Cytokine-cytokine receptor interaction	IL27-mediated signaling events	0.47619
HIF-1 signaling pathway	HIF-Fluid shear stress and atherosclerosis-alpha transcription factor network	0.47500
IL-17 signaling pathway	VEGF Hypoxia and Angiogenesis	0.46875
IL-17 signaling pathway	HIF-Fluid shear stress and atherosclerosis-alpha transcription factor network	0.46875
IL-17 signaling pathway	PI3K-Akt signaling pathway	0.46667
IL-17 signaling pathway	Free Radical Induced Apoptosis	0.45833
Ensemble of genes encoding ECM-associated proteins including ECM-affilaited proteins ECM regulators and secreted factors	Adhesion and Diapedesis of Granulocytes	0.45833
Focal adhesion	Cytokines can induce activation of matrix metalloproteinases which degrade extracellular matrix	0.45833
HIF-1 signaling pathway	Antifolate resistance	0.45833
	Angiopoietin receptor Tie2-mediated signaling	0.45395
Fluid shear stress and atherosclerosis		
Fluid shear stress and atherosclerosis	Glypican 1 network	0.44444
Fluid shear stress and atherosclerosis	Glypican 1 network	0.42424
Fluid shear stress and atherosclerosis	IL-17 signaling pathway	0.41071
Fluid shear stress and atherosclerosis	Cytokines can induce activation of matrix metalloproteinases which degrade extracellular matrix	0.40476
Fluid shear stress and atherosclerosis	IL27-mediated signaling events	0.40476
Fluid shear stress and atherosclerosis	Antifolate resistance	0.40476
PI3K-Akt signaling pathway	Plasma membrane estrogen receptor signaling	0.40000
	IL23-mediated signaling events	0.40000
IL-17 signaling pathway	amb2 Integrin signaling	0.40000
Cytokine-cytokine receptor interaction	Glypican 1 network	0.40000
Cytokine-cytokine receptor interaction	Toll-like receptor signaling pathway	0.40000
Cytokine-cytokine receptor interaction	Free Radical Induced Apoptosis	0.39583
Plasma membrane estrogen receptor signaling	Adhesion and Diapedesis of Granulocytes	0.39583
Cells and Molecules involved in local acute inflammatory response	Cytokines can induce activation of matrix metalloproteinases which degrade extracellular matrix	0.39583
Fat digestion and absorption	Antifolate resistance	0.39583
lntegrins in angiogenesis	Angiopoietin receptor Tie2-mediated signaling	0.38596
lntegrins in angiogenesis	amb2 Integrin signaling	0.38596
amb2 Integrin signaling	ABC transporters	0.38596
Mechanism of Gene Regulation by Peroxisome Proliferators via PPARa (alpha)	VEGF Hypoxia and Angiogenesis	0.38596
Free Radical Induced Apoptosis	HIF-Fluid shear stress and atherosclerosis-alpha transcription factor network	0.38596
	Angiopoietin receptor Tie2-mediated signaling	0.38596
Adhesion and Diapedesis of Granulocytes	ATF-Fluid shear stress and atherosclerosis transcription factor network	0.38596
	Ensemble of genes encoding ECM-associated proteins including ECM-affilaited proteins ECM regulators and secreted factors	0.38596
Ensemble of genes encoding ECM-associated proteins including ECM-affilaited proteins ECM regulators and secreted factors	Ensemble of genes encoding ECM-associated proteins including ECM-affilaited proteins ECM regulators and secreted factors	0.38596
		0.38596
Cells and Molecules involved in local acute inflammatory response	IL27-mediated signaling events	
IL23-mediated signaling events		0.38596
amb2 Integrin signaling	Toll-like receptor signaling pathway	
	Ensemble of genes encoding ECM-associated proteins including ECM-affilaited proteins ECM regulators and secreted factors	0.32500
Cells and Molecules involved in local acute inflammatory response	Ensemble of genes encoding ECM-associated proteins including ECM-affilaited proteins ECM regulators and secreted factors	0.37500
		0.31111
HIF-1 signaling pathway	IL-17 signaling pathway	0.37500
HIF-1 signaling pathway	HIF-Fluid shear stress and atherosclerosis-alpha transcription factor network	
Cytokine-cytokine receptor interaction	Angiopoietin receptor Tie2-mediated signaling	0.36111
Cytokine-cytokine receptor interaction	Toll-like receptor signaling pathway	0.33333
Plasma membrane estrogen receptor signaling		
IL23-mediated signaling events	Th17 cell differentiation	0.33333
IL23-mediated signaling events	amb2 Integrin signaling	0.33333
Mechanism of Gene Regulation by Peroxisome Proliferators via PPARa(alpha)	Mechanism of Gene Regulation by Peroxisome Proliferators via PPARa(alpha)	0.33333
	Hematopoietic cell lineage	0.32500
Fluid shear stress and atherosclerosis	Hematopoietic cell lineage	0.32500
PI3K-Akt signaling pathway	HIF-Fluid shear stress and atherosclerosis-alpha transcription factor network	0.32500
IL23-mediated signaling events	Angiopoietin receptor Tie2-mediated signaling	0.32500
IL23-mediated signaling events	Toll-like receptor signaling pathway	0.31667
Toll-like receptor signaling pathway	Th17 cell differentiation	0.31250
Cytokine-cytokine receptor interaction	Hematopoietic cell lineage	0.31111
Fluid shear stress and atherosclerosis	Cells and Molecules involved in local acute inflammatory response	0.31111
Cells and Molecules involved in local acute inflammatory response	Cytokine-cytokine receptor interaction	0.31111
	Ensemble of genes encoding ECM-associated proteins including ECM-affilaited proteins ECM regulators and secreted factors	0.30882
		0.27143
IL-17 signaling pathway	amb2 Integrin signaling	0.30000
IL-17 signaling pathway	Hematopoietic cell lineage	
Ensemble of genes encoding ECM-associated proteins including ECM-affilaited proteins ECM regulators and secreted factors	ATF-Fluid shear stress and atherosclerosis transcription factor network	0.30000
Fluid shear stress and atherosclerosis		0.30000
	Ensemble of genes encoding ECM-associated proteins including ECM-affilaited proteins ECM regulators and secreted factors	0.30000
Fluid shear stress and atherosclerosis		
Phagosome	Focal adhesion	0.25758
	Fat digestion and absorption	0.28846
Phagosome	Hematopoietic cell lineage	
HIF-1 signaling pathway	Plasma membrane estrogen receptor signaling	0.28750
HIF-1 signaling pathway	lntegrins in angiogenesis	0.28333
HIF-1 signaling pathway		
Ensemble of genes encoding core extracellular matrix including ECM glycoproteins collagens and proteoglycans	Mineral absorption	0.28333
	Focal adhesion	0.27692
Plasma membrane estrogen receptor signaling		
		0.27692
Genes encoding enzymes and their regulators involved in the remodeling of the extracellular matrix	Genes encoding enzymes and their regulators involved in the remodeling of the extracellular matrix	0.27692
	amb2 Integrin signaling	0.27574
Ensemble of genes encoding core extracellular matrix including ECM glycoproteins collagens and proteoglycans		
Fluid shear stress and atherosclerosis	lntegrins in angiogenesis	0.27143
		0.27143
Fluid shear stress and atherosclerosis	lntegrins in angiogenesis	
Cytokine-cytokine receptor interaction	Mineral absorption	0.26667
Plasma membrane estrogen receptor signaling	Hematopoietic cell lineage	
IL-17 signaling pathway	Focal adhesion	0.26250
Cytokine-cytokine receptor interaction	PI3K-Akt signaling pathway	0.26250
	Th17 cell differentiation	0.26250
Cytokine-cytokine receptor interaction	Ensemble of genes encoding ECM-associated proteins including ECM-affilaited proteins ECM regulators and secreted factors	0.26250
Cytokine-cytokine receptor interaction		0.25882
	lntegrins in angiogenesis	0.12795
		0.25758
Plasma membrane estrogen receptor signaling	Hematopoietic cell lineage	.25595
Phagosome	Ensemble of genes encoding ECM-associated proteins including ECM-affilaited proteins ECM regulators and secreted factors	
Fluid shear stress and atherosclerosis	Toll-like receptor signaling pathway	0.25556
Fluid shear stress and atherosclerosis	Toll-like receptor signaling pathway	0.25556
Cytokine-cytokine receptor interaction	HIF-1 signaling pathway	0.24762
PI3K-Akt signaling pathway	PI3K-Akt signaling pathway	
Toll-like receptor signaling pathway	Toll-like receptor signaling pathway	0.24359
	Ensemble of genes encoding ECM-associated proteins including ECM-affilaited proteins ECM regulators and secreted factors	0.22549
PI3K-Akt signaling pathway		0.22500
Fluid shear stress and atherosclerosis	Ensemble of genes encoding core extracellular matrix including ECM glycoproteins collagens and proteoglycans	0.22286
Fluid shear stress and atherosclerosis	PI3K-Akt signaling pathway	0.22222
HIF-1 signaling pathway	Focal adhesion	0.21212
Ensemble of genes encoding ECM-associated proteins including ECM-affilaited proteins ECM regulators and secreted factors	Phagosome	0.58333
PI3K-Akt signaling pathway	Focal adhesion	0.19022
		0.17788
PI3K-Akt signaling pathway	Ensemble of genes encoding ECM-associated proteins including ECM-affilaited proteins ECM regulators and secreted factors	0.17763
		0.16993
HIF-1 signaling pathway	Phagosome	0.16667
Complement and coagulation cascades	Cytokine-cytokine receptor interaction	
HIF-1 signaling pathway	Genes encoding enzymes and their regulators involved in the remodeling of the extracellular matrix	0.15887
HIF-1 signaling pathway	Ensemble of genes encoding ECM-associated proteins including ECM-affilaited proteins ECM regulators and secreted factors	0.15873
Fluid shear stress and atherosclerosis	Genes encoding enzymes and their regulators involved in the remodeling of the extracellular matrix	
	Ensemble of genes encoding ECM-associated proteins including ECM-affilaited proteins ECM regulators and secreted factors	0.14348
Complement and coagulation cascades		0.51136
		0.14091
		0.13846
		0.13636
		0.12795

### Identification of genes related to AMD

**FIGURE 5:**
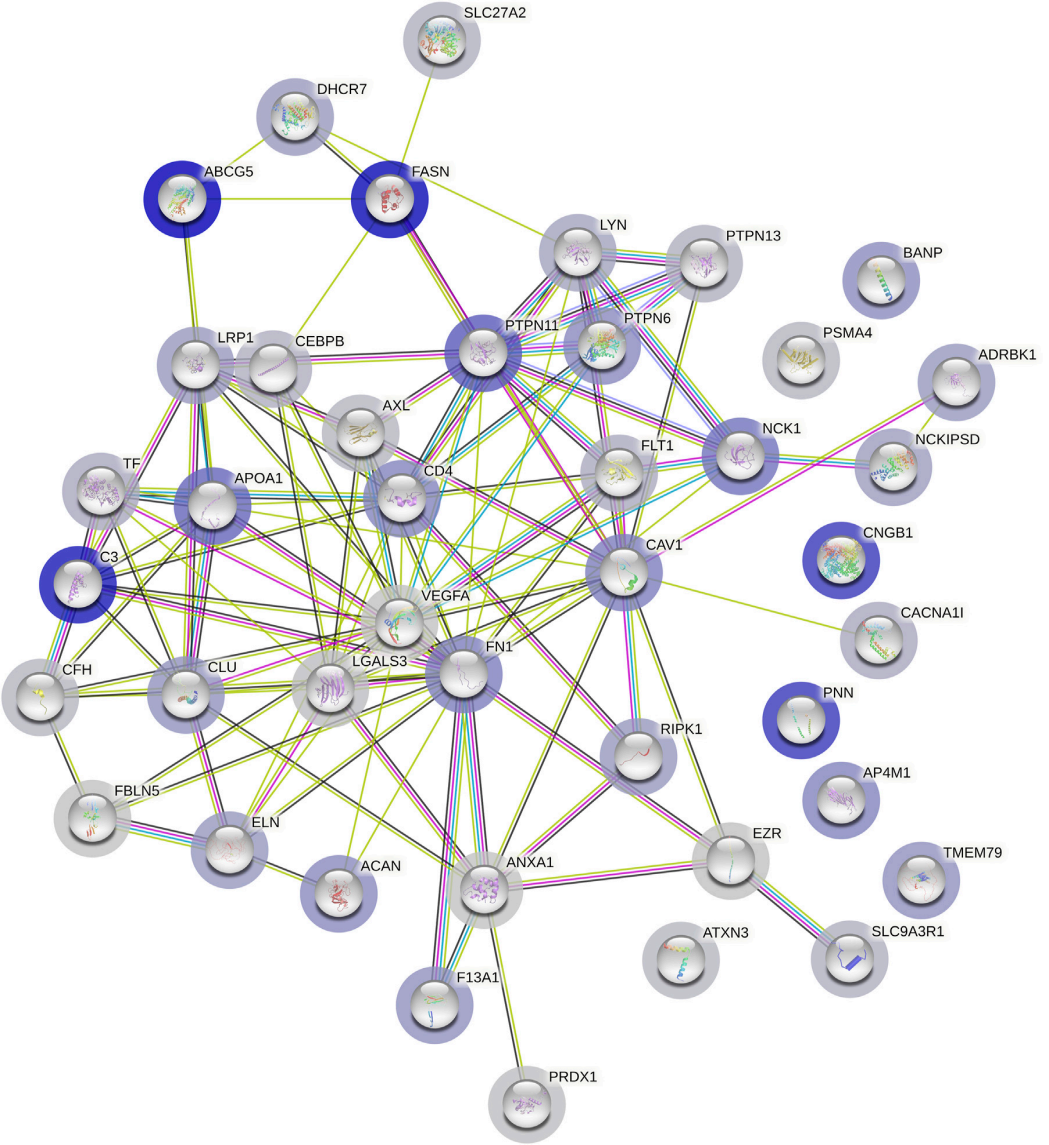
Protein-protein interaction network of the 42 genes in AMD gene collection. The blue halo around the gene indicates high betweenness while the gray halo indicates low betweenness.

**FIGURE 6:**
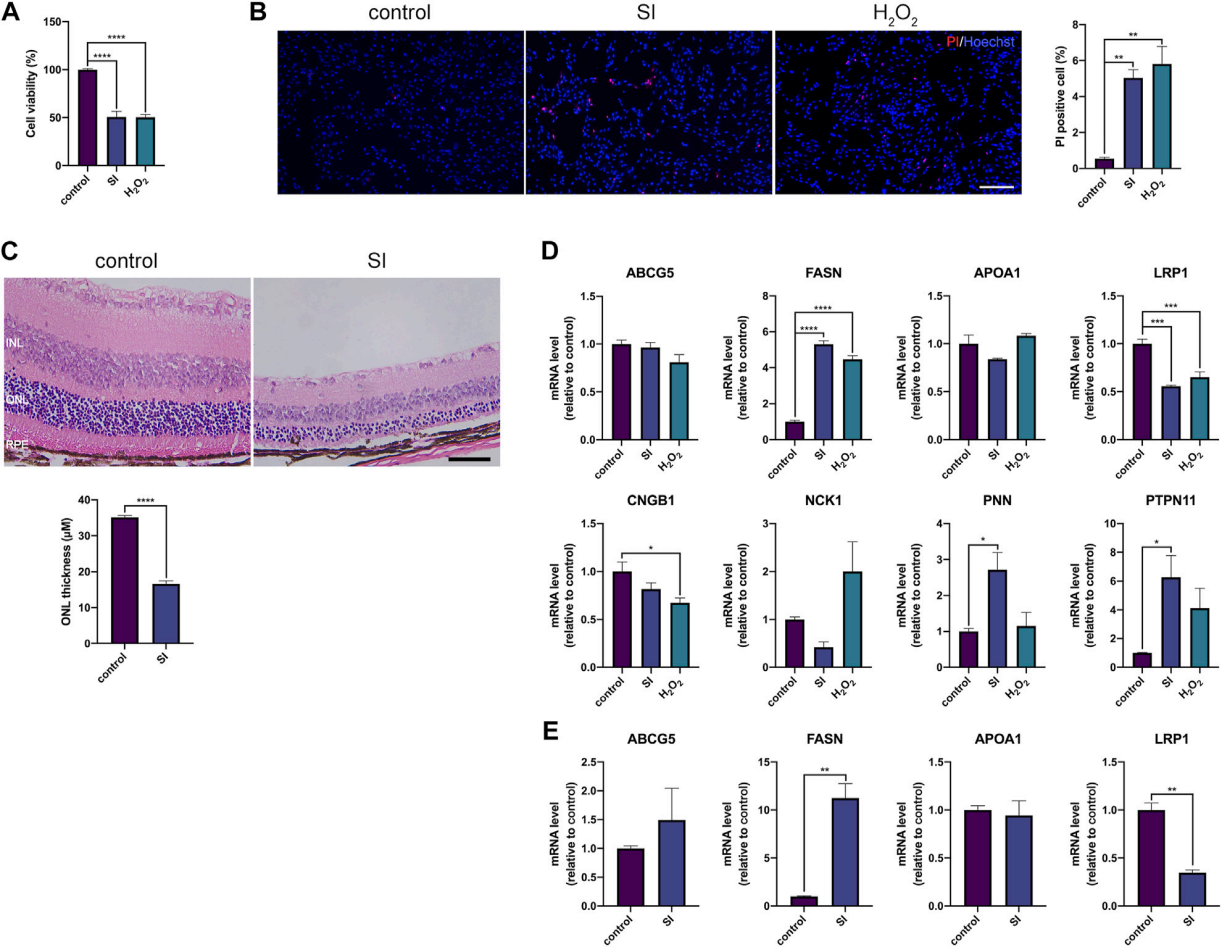
**(A)** CCk-8 results of RPE cells that were under SI (40 mM) or H_2_O_2_ (200 μM) treatment for 4 h **(B)** Representative images and the corresponding statistical result of PI staining. The cells were under SI (40 mM) or H_2_O_2_ (200 μM) treatment for 4 h; scale bar = 200 μm **(C)** H&E staining of retinal sections from mice at 2 days after 40 mg/kg SI injection; scale bar = 50 μm **(D)** Quantification of mRNA expression of indicated genes in RPE cells. The cells were under SI (40 mM) or H_2_O_2_ (200 μM) treatment for 4 h **(E)** Quantification of mRNA expression of indicated genes in RPE-choroid complex in mouse that were treated with SI for 2 days **p < 0.01, ***p < 0.001, ****p < 0.0001, compared versus control. INL: inner nuclear layer, ONL: outer nuclear layer, RPE: retinal pigmented epithelium.

To make a more comprehensive list of AMD related genes, we used shortest path algorithm based on the background human interactome which contained 13,460 nodes and 138,427 edges and provided by a recent study (Menche et al., 2015). The primary analysis extracted 4,587 genes participated in AMD protein-protein interaction (PPI) network. We discarded genes of which the betweenness was below 1,000 and conducted permutation test. Finally, in our collection, we obtained 42 genes highly associated with AMD (Table 5). The PPI network among the 42 genes were showed in Figure 5. There were 7 genes belonged to AMDgset, including C3, ELN, TF, FLT1, CFH, VEGFA and FBLN5 (Stone et al., 2004; Fang et al., 2009; Anderson et al., 2010; Yamashiro et al., 2011; Wysokinski et al., 2013; Owen et al., 2014), indicating our results identified many novel genes that are potentially associated with AMD. The genes associated with lipid metabolism had high betweenness, such as ABCG5, FASN, APOA1, and LRP1. Han et al., reported that higher APOA1 level increased the risk of AMD (Han et al., 2021). Since these genes were not included in the AMDgset, we intended to make a brief validation on their potential in further investigation of AMD. We used sodium iodate (SI) and H_2_O_2_ to treat RPE cells and establish an AMD cell model (Elliot et al., 2006; Tao et al., 2013). Moreover, we used SI to induce an AMD mouse model (Carido et al., 2014)Hanus, 2016 #2412}. The results of CCK-8 and PI staining confirmed RPE cell death and indicated that the AMD cell model was successfully established (Figures 6A,B). The results of H&E staining showed the AMD-like phenotype in the retina of the mouse under SI treatment (Figure 6C). Then we evaluated the mRNA levels of several genes with high betweenness including ABCG5, FASN, APOA1, LRP1, CNGB1, NCK1, PNN1, and PTPN11. The qRT-PCR results showed that FASN was up-regulated while LRP1 was downregulated in AMD cell and mouse model (Figures 6D,E). Storck et al., reported that selective deletion of LRP1 in the brain endothelium of C57BL/6 mice strongly reduced brain efflux of injected Aβ (1–42) (Storck et al., 2016). Since Aβ is also a crucial component of drusen, our results suggest that the downregulation of LRP1 might promote drusen formation in AMD. The function of FASN is to promote saturated fatty acid (SFA) synthesis. Previous study confirmed that SFA was associated significantly with increased risk of AMD (Agron et al., 2021). Therefore, the upregulation of FASN might exert a pro-AMD effect through promoting SFA synthesis. The mRNA levels of ABCG5 and APOA1 were relatively low in RPE cells and were not significantly altered (Figures 6D,E). We speculated that these genes might participate in AMD pathogenesis by acting in other tissues such as liver or intestine where they modulate fat digestion and absorption. Moreover, besides genes associated with lipid metabolism, some other genes in our collection were reported to participate in AMD progression or therapy e.g. NCK1 and EZR (Murad et al., 2014; Dubrac et al., 2016). The mRNA level of NCK1 was upregulated in the H_2_O_2_ AMD cell model (Figure 6D). Previous study showed that NCK1 knockdown was associated with neovascular inhibition (Dubrac et al., 2016). However, the mRNA level of NCK1 was slightly decreased in the SI AMD cell model, the reason might be different damage mode between SI and H_2_O_2_ PTPN11 was reported to be a diagnostic marker of AMD (Li et al., 2022). We also detected a significant upregulation of PTPN11 in the SI AMD cell model, indicating a potential role of PTPN11 in RPE degeneration. The exact role of NCK1 and PTPN11 in AMD progression needs further investigation in more AMD models. These results confirmed that our novel AMD gene collection have significant importance in guiding further investigation on AMD.

**TABLE 5:** Shortest path genes with betweenness greater than 1,000.

Gene ID	Official Symbol	Official Full Name	Betweenness
64240	ABCG5	ATP binding cassette subfamily G member 5	5123
2194	FASN	fatty acid synthase	4885
718	C3^a^	complement C3	4533
1258	CNGB1	cyclic nucleotide gated channel beta 1	3931
5411	PNN	pinin, desmosome associated protein	3892
5781	PTPN11	protein tyrosine phosphatase, non-receptor type 11	3207
335	APOA1	apolipoprotein A1	2980
4690	NCK1	NCK adaptor protein 1	2640
857	CAV1	caveolin 1	2468
2335	FN1	fibronectin 1	2421
9179	AP4M1	adaptor related protein complex 4 subunit mu 1	2330
920	CD4	CD4 molecule	2310
5777	PTPN6	protein tyrosine phosphatase, non-receptor type 6	2248
176	ACAN	aggrecan	2218
54971	BANP	BTG3 associated nuclear protein)	2118
84283	TMEM79	transmembrane protein 79	2100
2162	F13A1	coagulation factor XIII A chain	2073
1191	CLU	clusterin	2002
2006	ELN^a^	elastin	1926
156	GRK2	G protein-coupled receptor kinase 2	1911
8737	RIPK1	receptor interacting serine/threonine kinase 1	1899
4035	LRP1	LDL receptor related protein 1	1885
1717	DHCR7	7-dehydrocholesterol reductase	1862
51517	NCKIPSD	NCK interacting protein with SH3 domain	1843
4067	LYN	LYN proto-oncogene, Src family tyrosine kinase	1698
7018	TF^a^	transferrin	1591
8911	CACNA1I	calcium voltage-gated channel subunit alpha1 I	1580
2321	FLT1a	fms related tyrosine kinase 1	1501
1051	CEBPB	CCAAT/enhancer binding protein beta	1458
5783	PTPN13	protein tyrosine phosphatase, non-receptor type 13	1426
9368	SLC9A3R1	SLC9A3 regulator 1	1413
11001	SLC27A2	solute carrier family 27 member 2	1411
5685	PSMA4	proteasome subunit alpha 4	1342
3075	CFH^a^	complement factor H	1323
558	AXL	AXL receptor tyrosine kinase	1289
4287	ATXN3	ataxin 3	1261
3958	LGALS3	galectin 3	1143
5052	PRDX1	peroxiredoxin 1	1112
7430	EZR	ezrin	1083
7422	VEGFA^a^	vascular endothelial growth factor A	1056
10516	FBLN5^a^	fibulin 5	1045
301	ANXA1	annexin A1	1044

a Genes included in AMDgset.

## Discussion

Studies have confirmed that there is a strong correlation between a family history of AMD and the subsequent development of both dry and wet form of the disease. Genetic factors play a potential role in the etiology of AMD, explaining 46%–71% of the variation in the overall severity of the disease, while environmental factors take charge of the rest (Seddon et al., 2005). According to Yu et al., we only have recognized half of genetic risk factors of AMD (Yu et al., 2011). Therefore, making predictions based on the identified genetic risk factors and a comprehensive human interactome could be valuable to take a glimpse into the unknown half. A previous study about AMD related GO analysis showed a variant result with ours as they found the most significant terms are plasma membrane, cell surface receptor linked signal transduction and intracellular signaling cascade (Zhang et al., 2013). The inconformity between our results may ascribe to the method we chose genes and the quantity of genes we retrieved. In our study, we firstly established a relatively comprehensive collection of the genes genetically associated with AMD. Then, we proceeded GO enrichment and pathway enrichment analyses to demonstrate the most significant biological functions and cellular signaling related to AMD. Moreover, the results of crosstalk study showed a visualized interaction of pathways that we have identified. At last, we made a predictive list of potential AMD related genes by using shortest path algorithm and confirmed that FASN and LRP1 were potentially associated with AMD. By retrieving AMDgset from PUBMED, we obtained 176 genes which were reported significantly genetically related to AMD. Both dry and wet forms of AMD were included in our research. According to the clinical character of AMD, new vessels may invade the outer retina, subretinal space or subRPE space, resulting in macular neovascularization (MNV) at any stage of dry AMD (Fleckenstein et al., 2021). The natural course of AMD indicates that the pathogeneses of dry and wet AMD are common to a great extent. Therefore, it is of great significance to study the genetic risk factors and the pathway crosstalk in the combination of dry and wet AMD.

Our pathway analysis revealed that complement related pathway was enriched in AMDgset. This finding further consolidates the link between AMD and complement system. Precedent identification of several molecular components of the complement cascade in drusen suggests that complement activation is an important element in drusen biogenesis (Johnson et al., 2001). CFH binds to glycoaminoglycans (GAG) on host cells and apoptotic bodies and acts as a cofactor of Complement factor I (CFI) that cleaves C3b into iC3b and prevents membrane attack complex (MAC) formation (Atkinson and Goodship, 2007). Hageman et al. demonstrated that risk alleles decreased the function of CFH, which may lead to high MAC aggregation at the RPE-choroid interface and jeopardize the integrity of Bruch’s membrane (Hageman et al., 2005). However, Hageman et al. claimed that CFH immunoreactivity in the eye is stronger, not weaker, in AMD donor tissues. Calippe et al. recently showed that the AMD-associated CFH variant CFH(H402) contributes to AMD etiology by increasing subretinal macrophage accumulation through binding CD11b. Together with their results, there is a discrepancy with the function of CFH in AMD progression that need to be well studied in the future. Cipriani et al. recently revealed that AMD was associated with genetically driven elevated circulating levels of complement factor H related 4 (CFHR4). The role of complement factor H related 1 (CFHR1) is protecting intercapillary septa ECM from complement activation (Clark et al., 2014; McHarg et al., 2015), but this protective function may be diminished by elevation of CFHR4. Strong evidences indicate that these abnormities result in dysregulation of the complement cascade and aberrant activation of the immune system. Besides, we noticed that pathways associated with hypoxia and angiogenesis were also enriched in AMDgset. The mechanism may due to the limited blood supply which is caused by choroidal capillary atrophy and high oxygen demand in macula. This imbalance situation causes relative hypoxia, which furtherly up-regulates the expression of growth factors, such as VEGF family (Penfold et al., 2001).

In our pathway crosstalk analysis, we demonstrated two main components interacted with each other. One component was mainly predominated by inflammation related pathways while another was hypoxia-angiogenesis related pathways. The two modules were connected by cytokine-cytokine receptor interaction pathway (genes: TLR4, NOS2, NOS3, VEGFA) instead of operating separately. We attach much importance to the mediating role of cytokines-cytokine receptors signaling and speculate that the cytokines and chemokines related to macrophages, RPE cells and vessel endothelial cells play a central role in mediating two main modules of AMD associated pathways. TLR2/TLR4 plays a prominent role in recognizing pathogen-associated molecular pattern (PAMP) or damage-associated molecular patterns (DAMP) and activates NLRP3 inflammasome or NF-κB related pathways to modulate inflammation state (Schmitz and Orso, 2002; Allan et al., 2005; Schroder and Tschopp, 2010). The pro-inflammation, anti-angiogenic, potentially neurotoxic state is characterized by IL-1β, TNF-α, IL-6, CCL2 and iNOS, while the anti-inflammation, wound healing, fibrosis state is defined by VEGF, IL-10 and IL-1RA among others (Sica and Mantovani, 2012; Wynn and Vannella, 2016). It is interesting that our pathway crosstalk analysis also reflected this phenomenon. The larger module contained the acute inflammatory response and ECM degradation pathways, which indicated the pro-inflammatory state. Those potentially neurotoxic cytokines may contribute to RPE and photoreceptor degeneration and result in the geographic atrophy. The smaller module contained angiogenesis pathways, which indicated the anti-inflammatory state and CNV formation. Our pathway crosstalk study is of great significance as it reflects the pivotal role of cytokines and cytokine receptors in prompting early AMD to the two distinct types. It also indicated that there might be a possibility to modulate the specific type of cytokines in early AMD to control its progression. There are limited researches focused on the role of TLR4 and NOS family in AMD. Chen et al. demonstrated that TLR4 mediated subretinally-deposited amyloid-β induced angiogenic and inflammation (Chen et al., 2016). Imran A. Bhutto et al. showed that the decrease in retinal NOS1 in AMD eyes was probably related to neuronal degeneration. The decrease in NOS1 and NOS3 in AMD choroid could be associated with vasoconstriction and hemodynamic changes (Bhutto et al., 2010). We strongly propose that future studies should focus on these cytokines and cytokine receptors.

In our novel gene collection, besides the genes we have verified, CNGB1 is also a candidate gene that might participate AMD. CNGB1 is a gene encoding cyclic nucleotide-gated (CNG) channels proteins which are key components for signal transduction in rod outer segment and olfactory sensory neurons (OSNs) (Charbel Issa et al., 2018). It has been verified that AMD patients suffer from impaired dark adaptation, which indicates a rod deficiency (Flamendorf et al., 2015). Zhang et al. found that the amplitude of dark adaptive b-wave was significantly diminished in CNGB1 knockout mice, more importantly, these mice showed a rod-cone degeneration. These results strongly implicate that CNGB1 may account for the deteriorated dark adaptation in AMD especially in the dry form. Although the mRNA level of CNGB1 is decreased only in H_2_O_2_ AMD cell model, considering the fact that the cell model was established by RPE cells, further study should investigate the dysregulation of CNGB1 in photoreceptor cells in AMD model.

Although we have provided a new perspective on AMD associated genes, there are several limitations of our study. First, most of our results are based on literatures, so the partialness of some studies can affect our analysis. Second, the identification of AMD risk genes is a gradual process, as well as the background human interactome. The incomplete human interactome may bring some false-positive or false-negative results to our study. More importantly, the genes in our novel collection should be verified in more cell models and animal models of AMD.

## Conclusion

Our study filled the gap in the integrated study in genetic field of AMD, and we revealed the potential relationships between these pathways as well as their operation pattern. Moreover, we demonstrated a relatively comprehensive AMD associated genes list and validated that the mRNA levels of FASN and LRP1 are dysregulated in both cell and mouse models of AMD, indicating they might regulate AMD progression directly.

## Data Availability

The original contributions presented in the study are included in the article/Supplementary Material, further inquiries can be directed to the corresponding author.
