# Supplementary material for: Potential gene identification and pathway crosstalk analysis of age-related macular degeneration
Source: Front Genet. 2022 Sep 6;13:992328. doi: 10.3389/fgene.2022.992328 (PMC9486309; doi:10.3389/fgene.2022.992328)
Supplement: Supplementary file 1 [file Table1.DOCX]

| Table1. Genes retrieved from human genetic association studies | | | | |
| --- | --- | --- | --- | --- |
| Gene Symbol | Gene ID | | Full Name | |
| ABCG1  ABCG8  ABHD2  ACAD10  ACE  ADAMTS9  ALDH3A2  ANGPT2  APOE  ARHGAP21  ARMS2  ASPM  B3GLCT  BCO1  BCO2  C2  C20orf85  C3  C4A  C6orf223  C9  CACNG3  CAPN5  CATSPER2  CCL2  CCR2  CCR3  CD36  CD63  CETP  CFB  CFD  CFH  CFHR1  CFHR2  CFHR3  CFHR4  CFHR5  CFI  CLUL1  CNN2  COL10A1  COL15A1  COL4A3  COL8A1  CRP  CST3  CTRB1  CTRB2  CX3CR1  CXCL8  CYP1A2  CYP2R1  CYP46A1  DAPL1  DDR1  ELN  ELOVL4  ERCC2  ERCC6  ESR1  F13B  FADS1  FADS2  FBLN5  FCGR2A  FGD6  FGL1  FILIP1L  FKBPL  FLT1  FPR1  FRK  GAS6  GPX1  GPX3  GRK5  GSTM1  HLA-B  HLA-C  HLA-DQB1  HMCN1  HMOX1  HMOX2  HTRA1  IER3  IGF1R  IL17A  IL17RC  IL1B  KCTD10  KDR  KMT2E  LIPC  LOXL1  LRP6  MALL  MMP2  MMP20  MMP9  MRPL10  MT2A  MTHFR  MTR  MYRIP  NFE2L2  NOS2  NOS3  NPC1L1  NPHP1  NPLOC4  NQO1  OSBP2  P2RX4  P2RX7  PGF  PILRA  PILRB  PLEKHA1  PON1  PPARG  PPARGC1A  PRKDC  PRKN  PRLR  PTCHD3  RAD51  RAD51B  RDH5  RGS10  RHO  RLBP1  ROBO1  RORA  RORB  RXRA  SCARB1  SELP  SERPINF1  SERPING1  SIRT1  SKIV2L  SLC16A8  SLC44A4  SMUG1  SOD2  SOD3  SPEF2  SRPK2  STRC  SYN3  TF  TFR2  TFRC  TGFBR1  TIMP3  TLR2  TLR3  TLR4  TMEM97  TNF  TNFRSF10A  TNMD  TNXB  TRPM1  TRPM3  TSPAN10  UBE3D  UNG  VDR  VEGFA  VLDLR  VTN  ZBTB41 | | 9619  64241  11057  80724  1636  56999  224  285  348  57584  387715  259266  145173  53630  83875  717  128602  718  720  221416  735  10368  726  117155  6347  729230  1232  948  967  1071  629  1675  3075  3078  3080  10878  10877  81494  3426  27098  1256  1300  1306  1285  1295  1401  1471  1504  440387  13051  3576  1544  120227  10858  92196  780  2006  6785  2068  2074  2099  2165  3992  9415  10516  2212  55785  2267  11259  63943  2321  2357  2444  2621  2876  2878  2869  2944  3106  3017  3119  83872  3162  3163  5654  8870  3480  3605  84818  3553  83892  3791  55904  3990  4016  4040  7851  4313  9313  4318  124995  4502  4524  4548  25924  4780  4843  4846  29881  4867  55666  1728  23762  5025  5027  5228  29992  29990  59338  5444  5468  10891  5591  5071  5618  374308  5888  5890  5959  6001  6010  6017  6091  6095  6096  6256  949  6403  5176  710  23411  6499  23539  80736  23583  6648  6649  79925  6733  161497  8224  7018  7036  7037  7046  7078  7097  7098  7099  27346  7124  8797  64102  7148  4308  80036  83882  90025  7374  7421  7422  7436  7448  226470 | | ATP binding cassette subfamily G member 1  ATP binding cassette subfamily G member 8  abhydrolase domain containing 2  acyl-CoA dehydrogenase family member 10  angiotensin I converting enzyme  ADAM metallopeptidase with thrombospondin type 1 motif 9  aldehyde dehydrogenase 3 family member A2  angiopoietin 2  apolipoprotein E  Rho GTPase activating protein 21  age-related maculopathy susceptibility 2  abnormal spindle microtubule assembly  beta 3-glucosyltransferase  beta-carotene oxygenase 1  beta-carotene oxygenase 2  complement C2  chromosome 20 open reading frame 85  complement C3  complement C4A (Rodgers blood group)  chromosome 6 open reading frame 223  complement C9  calcium voltage-gated channel auxiliary subunit gamma 3  calpain 5  cation channel sperm associated 2  C-C motif chemokine ligand 2  C-C motif chemokine receptor 2  C-C motif chemokine receptor 3  CD36 molecule  CD63 molecule  cholesteryl ester transfer protein  complement factor B  complement factor D  complement factor H  complement factor H related 1  complement factor H related 2  complement factor H related 3  complement factor H related 4  complement factor H related 5  complement factor I  clusterin like 1  calponin 2  collagen type X alpha 1 chain  collagen type XV alpha 1 chain  collagen type IV alpha 3 chain  collagen type VIII alpha 1 chain  C-reactive protein [*Homo sapiens*  cystatin C  chymotrypsinogen B1  chymotrypsinogen B2  chemokine (C-X3-C motif) receptor 1  C-X-C motif chemokine ligand 8  cytochrome P450 family 1 subfamily A member 2  cytochrome P450 family 2 subfamily R member 1  cytochrome P450 family 46 subfamily A member 1  death associated protein like 1  discoidin domain receptor tyrosine kinase 1  elastin  ELOVL fatty acid elongase 4  ERCC excision repair 2, TFIIH core complex helicase subunit  ERCC excision repair 6, chromatin remodeling factor  estrogen receptor 1  coagulation factor XIII B chain  fatty acid desaturase 1  fatty acid desaturase 2  fibulin 5  Fc fragment of IgG receptor IIa  FYVE, RhoGEF and PH domain containing 6  fibrinogen like 1  filamin A interacting protein 1 like  FK506 binding protein like  fms related tyrosine kinase 1  formyl peptide receptor 1  fyn related Src family tyrosine kinase  growth arrest specific 6  glutathione peroxidase 1  glutathione peroxidase 3  G protein-coupled receptor kinase 5  glutathione S-transferase mu 1  major histocompatibility complex, class I, B  major histocompatibility complex, class I, C  major histocompatibility complex, class II, DQ beta 1  hemicentin 1  heme oxygenase 1  heme oxygenase 2  HtrA serine peptidase 1  immediate early response 3  insulin like growth factor 1 receptor  interleukin 17A  interleukin 17 receptor C  interleukin 1 beta  potassium channel tetramerization domain containing 10  kinase insert domain receptor  lysine methyltransferase 2E  lipase C, hepatic type  lysyl oxidase like 1  LDL receptor related protein 6  mal, T cell differentiation protein like  matrix metallopeptidase 2  matrix metallopeptidase 20  matrix metallopeptidase 9  mitochondrial ribosomal protein L10  metallothionein 2A  methylenetetrahydrofolate reductase  5-methyltetrahydrofolate-homocysteine methyltransferase  myosin VIIA and Rab interacting protein  nuclear factor, erythroid 2 like 2  nitric oxide synthase 2  nitric oxide synthase 3  NPC1 like intracellular cholesterol transporter 1  nephrocystin 1  NPL4 homolog, ubiquitin recognition factor  NAD(P)H quinone dehydrogenase 1  oxysterol binding protein 2  purinergic receptor P2X 4  purinergic receptor P2X 7  placental growth factor  paired immunoglobin like type 2 receptor alpha  paired immunoglobin like type 2 receptor beta  pleckstrin homology domain containing A1  paraoxonase 1  peroxisome proliferator activated receptor gamma  PPARG coactivator 1 alpha  protein kinase, DNA-activated, catalytic polypeptide  parkin RBR E3 ubiquitin protein ligase  prolactin receptor  patched domain containing 3  RAD51recombinase  RAD51 paralog B  retinol dehydrogenase 5  regulator of G protein signaling 10  rhodopsin [Homo sapiens  retinaldehyde binding protein 1  roundabout guidance receptor 1  RAR related orphan receptor A  RAR related orphan receptor B  retinoid X receptor alpha  scavenger receptor class B member 1  selectin P  serpin family F member 1  serpin family G member 1  sirtuin 1  Ski2 like RNA helicase  solute carrier family 16 member 8  solute carrier family 44 member 4  single-strand-selective monofunctional uracil-DNA glycosylase  superoxide dismutase 2  superoxide dismutase 3  sperm flagellar 2  SRSF protein kinase 2  stereocilin  synapsin III  transferrin  transferrin receptor 2  transferrin receptor  transforming growth factor beta receptor 1  TIMP metallopeptidase inhibitor 3  toll like receptor 2  toll like receptor 3  toll like receptor 4  transmembrane protein 97  tumor necrosis factor  TNF receptor superfamily member 10a  tenomodulin  tenascin XB  transient receptor potential cation channel subfamily M member 1  transient receptor potential cation channel subfamily M member 3  tetraspanin 10  ubiquitin protein ligase E3D  uracil DNA glycosylase  vitamin D receptor  vascular endothelial growth factor A  very low density lipoprotein receptor  vitronectin  zinc finger and BTB domain containing 41 |
