# Supplementary material for: Potential gene identification and pathway crosstalk analysis of age-related macular degeneration
Source: Front Genet. 2022 Sep 6;13:992328. doi: 10.3389/fgene.2022.992328 (PMC9486309; doi:10.3389/fgene.2022.992328)
Supplement: Supplementary file 2 [file Table2.DOCX]

| **Table 2.** Gene Ontology (GO) terms enriched with AMDgset (Top 10 terms) | | | |
| --- | --- | --- | --- |
| Go terms | *P*^a^ | *P*_BH_^b^ | Observed |
| *Molecular Function*  GO:0005102: signaling receptor binding  GO:0071814: protein-lipid complex binding  GO:0071813: lipoprotein particle binding  GO:0008289: lipid binding  GO:1901681: sulfur compound binding  GO:0017127: cholesterol transporter activity  GO:0060089: molecular transducer activity  GO:0038023: signaling receptor activity  GO:0034185: apolipoprotein binding  GO:0032934: sterol binding  *Biological Process*  GO:1901700: response to oxygen-containing compound  GO:0009611: response to wounding  GO:1903034: regulation of response to wounding  GO:0050727: regulation of inflammatory response  GO:0006954: inflammatory response  GO:0032101: regulation of response to external stimulus  GO:0033993: response to lipid  GO:0001525: angiogenesis  GO:0010035: response to inorganic substance  GO:0072593: reactive oxygen species metabolic process  *Cellular Component*  GO:0005615: extracellular space  GO:0009986: cell surface  GO:0031012: extracellular matrix  GO:0044420: extracellular matrix component  GO:0005578: proteinaceous extracellular matrix  GO:0009897: external side of plasma membrane  GO:0072562: blood microparticle  GO:0005604: basement membrane  GO:0098552: side of membrane  GO:0044433: cytoplasmic vesicle part | 5.783×10^-10^  2.408×10^-9^  2.408×10^-9^  1.89×10^-8^  1.019×10^-7^  1.045×10^-7^  1.247×10^-7^  1.571×10^-7^  2.246×10^-7^  2.282×10^-7^  5.695×10^-25^  3.818×10^-24^  8.509×10^-21^  8.855×10^-20^  1.297×10^-19^  5.007×10^-19^  4.416×10^-18^  4.654×10^-18^  1.413×10^-17^  3.054×10^-17^  5.038×10^-22^  6.597×10^-13^  2.024×10^-11^  4.704×10^-11^  3.289×10^-10^  5.808×10^-10^  7.148×10^-10^  5.02×10^-9^  5.07×10^-8^  5.919×10^-8^ | 4.835×10^-7^  6.711×10^-7^  6.711×10^-7^  3.949×10^-6^  1.455×10^-5^  1.455×10^-5^  1.49×10^-5^  1.642×10^-5^  1.823×10^-5^  1.823×10^-5^  2.764×10^-21^  9.267×10^-21^  1.377×10^-17^  1.075×10^-16^  1.259×10^-16^  4.051×10^-16^  2.824×10^-15^  2.824×10^-15^  7.622×10^-15^  1.482×10^-14^  2.081×10^-19^  1.362×10^-10^  2.786×10^-9^  4.857×10^-9^  2.717×10^-8^  3.998×10^-8^  4.217×10^-8^  2.592×10^-7^  2.327×10^-6^  2.444×10^-6^ | 41  7  7  24  14  5  38  34  5  7  64  50  34  29  39  45  44  31  33  24  57  34  23  14  20  18  13  11  20  22 |

AMDgset: age-related macular degeneration-related genes gene set

^a^*P* values were calculated by Fisher’s exact test

^b^*P*_BH_ values were adjusted by Benjamini and Hochberg (BH) method
