# Supplementary material for: Potential gene identification and pathway crosstalk analysis of age-related macular degeneration
Source: Front Genet. 2022 Sep 6;13:992328. doi: 10.3389/fgene.2022.992328 (PMC9486309; doi:10.3389/fgene.2022.992328)
Supplement: Supplementary file 3 [file Table5.DOCX]

| **Table 5.** Shortest path genes with betweenness greater than 1000 | | | | |
| --- | --- | --- | --- | --- |
| Gene ID | Official Symbol | Official Full Name | Betweenness |  |
| 64240  2194  718  1258  5411  5781  335  4690  857  2335  9179  920  5777  176  54971  84283  2162  1191  2006  156  8737  4035  1717  51517  4067  7018  8911  2321  1051  5783  9368  11001  5685  3075  558  4287  3958  5052  7430  7422  10516  301 | ABCG5  FASN  C3*  CNGB1  PNN  PTPN11  APOA1  NCK1  CAV1  FN1  AP4M1  CD4  PTPN6  ACAN  BANP  TMEM79  F13A1  CLU  ELN*  GRK2  RIPK1  LRP1  DHCR7  NCKIPSD  LYN  TF*  CACNA1I  FLT1*  CEBPB  PTPN13  SLC9A3R1  SLC27A2  PSMA4  CFH*  AXL  ATXN3  LGALS3  PRDX1  EZR  VEGFA*  FBLN5*  ANXA1 | ATP binding cassette subfamily G member 5  fatty acid synthase  complement C3  cyclic nucleotide gated channel beta 1  pinin, desmosome associated protein  protein tyrosine phosphatase, non-receptor type 11  apolipoprotein A1  NCK adaptor protein 1  caveolin 1  fibronectin 1  adaptor related protein complex 4 subunit mu 1  CD4 molecule  protein tyrosine phosphatase, non-receptor type 6  aggrecan  BTG3 associated nuclear protein)  transmembrane protein 79  coagulation factor XIII A chain  clusterin  elastin  G protein-coupled receptor kinase 2  receptor interacting serine/threonine kinase 1  LDL receptor related protein 1  7-dehydrocholesterol reductase  NCK interacting protein with SH3 domain  LYN proto-oncogene, Src family tyrosine kinase  transferrin  calcium voltage-gated channel subunit alpha1 I  fms related tyrosine kinase 1  CCAAT/enhancer binding protein beta  protein tyrosine phosphatase, non-receptor type 13  SLC9A3 regulator 1  solute carrier family 27 member 2  proteasome subunit alpha 4  complement factor H  AXL receptor tyrosine kinase  ataxin 3  galectin 3  peroxiredoxin 1  ezrin  vascular endothelial growth factor A  fibulin 5  annexin A1 | 5123  4885  4533  3931  3892  3207  2980  2640  2468  2421  2330  2310  2248  2218  2118  2100  2073  2002  1926  1911  1899  1885  1862  1843  1698  1591  1580  1501  1458  1426  1413  1411  1342  1323  1289  1261  1143  1112  1083  1056  1045  1044 |  |
| *Genes included in AMDgset | | | | |
