# Supplementary material for: Potential gene identification and pathway crosstalk analysis of age-related macular degeneration
Source: Front Genet. 2022 Sep 6;13:992328. doi: 10.3389/fgene.2022.992328 (PMC9486309; doi:10.3389/fgene.2022.992328)
Supplement: Supplementary file 4 [file Table3.DOCX]

| **Table 3.** Pathways enriched in AMDgset | | | |
| --- | --- | --- | --- |
| Pathways | *P*^a^ | *P*_BH_^b^ | Genes included in Pathways |
| Complement and coagulation cascades  Fluid shear stress and atherosclerosis  HIF-1 signaling pathway  Cytokine-cytokine receptor interaction  Plasma membrane estrogen receptor signaling  Cells and Molecules involved in local acute inflammatory response  PI3K-Akt signaling pathway  IL23-mediated signaling events  Phagosome  Ensemble of genes encoding core extracellular matrix including ECM glycoproteins, collagens and proteoglycans  Fat digestion and absorption  IL-17 signaling pathway  Genes encoding enzymes and their regulators involved in the remodeling of the extracellular matrix  Free Radical Induced Apoptosis  Integrins in angiogenesis  amb2 Integrin signaling  Mineral absorption  VEGF, Hypoxia, and Angiogenesis  Adhesion and Diapedesis of Granulocytes  Mechanism of Gene Regulation by Peroxisome Proliferators via PPARa(alpha)  Cytokines can induce activation of matrix metalloproteinases, which degrade extracellular matrix  The IGF-1 Receptor and Longevity  HIF-2-alpha transcription factor network  Toll-like receptor signaling pathway  Ensemble of genes encoding ECM-associated proteins including ECM-affilaited proteins, ECM regulators and secreted factors  **Table 3** (continued) | 2.404×10^-9^  1.532×10^-8^  3.59×10^-7^  8.867×10^-7^  1.274×10^-5^  4.264×10^-5^  6.555×10^-5^  7.585×10^-5^  1.009×10^-4^  1.221×10^-4^  1.254×10^-4^  1.277×10^-4^  1.426×10^-4^  1.933×10^-4^  2.186×10^-4^  2.679×10^-4^  3.57×10^-4^  3.803×10^-4^  5.66×10^-4^  6.509×10^-4^  7.013×10^-4^  7.013×10^-4^  7.087×10^-4^  1.593×10^-3^  1.749×10^-3^ | 4.712×10^-7^  2.002×10^-6^  1.716×10^-5^  3.476×10^-5^  2.628×10^-4^  7.268×10^-4^  1.028×10^-3^  1.144×10^-3^  1.465×10^-3^  1.668×10^-3^  1.668×10^-3^  1.668×10^-3^  1.803×10^-3^  2.368×10^-3^  2.521×10^-3^  3×10^-3^  3.782×10^-3^  3.822×10^-3^  5.283×10^-3^  5.934×10^-3^  6.039×10^-3^  6.039×10^-3^  6.039×10^-3^  1.224×10^-2^  1.292×10^-2^ | CFH, VTN, CFI, F13B, CFB, CFD, SERPING1, C2, C3, C4A, C9  HMOX1, HMOX2, GSTM1, NFE2L2, NQO1, CCL2, KDR, TNF, MMP2, MMP9, IL1B, NOS3, VEGFA  FLT1, ANGPT2, HMOX1, TF, TFRC, IGF1R, TLR4, NOS2, NOS3, VEGFA  FLT1, IL17A, IL17RC, TNFRSF10A, CCR2, TGFBR1, CCL2, KDR, CCR3, TNF, IL1B, PRLR, CX3CR1, CXCL8, VEGFA  ESR1, IGF1R, MMP2, MMP9, NOS3  SELP, C3, TNF, CXCL8  COL4A3, FLT1, VTN, ANGPT2, PGF, RXRA, IGF1R, TLR2, TLR4, KDR, TNXB, NOS3, PRLR, VEGFA  IL17A, CCL2, TNF, IL1B, NOS2  HLA-B, HLA-DQB1, TFRC, FCGR2A, CD36, SCARB1, TLR2, TLR4, C3  COL4A3, COL8A1, COL10A1, FBLN5, VTN, COL15A1, GAS6, KERA, HMCN1, ELN, FGL1, TNXB  ABCA1, CD36, SCARB1, NPC1L1, ABCG8  IL17A, IL17RC, CCL2, TNF, MMP9, IL1B, CXCL8  HTRA1, SERPINF1, MMP20, F13B, TIMP3, ADAMTS9, LOXL1, CST3, SERPING1, MMP2, MMP9  GPX1, TNF, CXCL8  COL4A3, VTN, IGF1R, KDR, VEGFA  SELP, VTN, TNF, MMP2, MMP9  HMOX1, HMOX2, TF, MT2A, VDR  FLT1, KDR, NOS3, VEGFA  SELP, TNF, CXCL8  RXRA, PPARGC1A, CD36, TNF, NOS2  ACE, TNF, IL1B  IGF1R, SOD2, SOD3  FLT1, SIRT1, KDR, VEGFA  TLR2, TLR3, TLR4, TNF, IL1B, CXCL8  IL17A, HTRA1, ANGPT2, SERPINF1, MMP20, PGF, F13B, TIMP3, ADAMTS9, LOXL1, CST3, SERPING1, CCL2, TNF, MMP2, MMP9, IL1B, CXCL8, VEGFA |
| Pathways | *P*^a^ | *P*_BH_^b^ | Genes included in Pathways |
| Th17 cell differentiation  Genes encoding collagen proteins  ABC transporters  Angiopoietin receptor Tie2-mediated signaling  Focal adhesion  Glypican 1 network  IL27-mediated signaling events  ATF-2 transcription factor network  Longevity regulating pathway  Protein digestion and absorption  Antifolate resistance  IL 5 Signaling Pathway  Hematopoietic cell lineage  Signaling mediated by p38-alpha and p38-beta | 1.842×10^-3^  1.89×10^-3^  2.055×10^-3^  2.61×10^-3^  2.815×10^-3^  3.637×10^-3^  3.637×10^-3^  4.287×10^-3^  4.378×10^-3^  4.592×10^-3^  6.018×10^-3^  6.09×10^-3^  6.298×10^-3^  8.462×10^-3^ | 1.313×10^-2^  1.323×10^-2^  1.389×10^-2^  1.734×10^-2^  1.839×10^-2^  2.263×10^-2^  2.263×10^-2^  2.626×10^-2^  2.64×10^-2^  2.727×10^-2^  3.511×10^-2^  3.511×10^-2^  3.578×10^-2^  4.672×10^-2^ | IL17A, HLA-DQB1, RXRA, TGFBR1, RORA, IL1B  COL4A3, COL8A1, COL10A1, COL15A1  ABCA1, ABCA4, ABCG1, ABCG8  ANGPT2, TNF, MMP2, NOS3  COL4A3, FLT1, VTN, PGF, IGF1R, KDR, TNXB, VEGFA  FLT1, TGFBR1, VEGFA  IL17A, TNF, IL1B  PPARGC1A, MMP2, NOS2, CXCL8  PPARG, SIRT1, PPARGC1A, IGF1R, SOD2  COL4A3, COL10A1, COL15A1, ELN, CTRB1  MTHFR, TNF, IL1B  CCR3, IL1B  HLA-DQB1, TFRC, CD36, TNF, IL1B  ESR1, PPARGC1A, NOS2 |

^a^*P* values were calculated by Fisher’s exact test

^b^*P*_BH_ values were adjusted by Benjamini and Hochberg (BH) method
