# Supplementary material for: Potential gene identification and pathway crosstalk analysis of age-related macular degeneration
Source: Front Genet. 2022 Sep 6;13:992328. doi: 10.3389/fgene.2022.992328 (PMC9486309; doi:10.3389/fgene.2022.992328)
Supplement: Supplementary file 5 [file Table4.DOCX]

| Table 4. Pathway crosstalk information | | | | |
| --- | --- | --- | --- | --- |
| Pathway A | Pathway B | | | Score |
| Cells and Molecules involved in local acute inflammatory response  lntegrins in angiogenesis  Genes encoding enzymes and their regulators involved in the remodeling of the extracellular matrix  IL23-mediated signaling events  PI3K-Akt signaling pathway  IL-17 signaling pathway  PI3K-Akt signaling pathway  VEGF Hypoxia and Angiogenesis  Ensemble of genes encoding core extracellular matrix including ECM glycoproteins collagens and proteoglycans  IL23-mediated signaling events  PI3K-Akt signaling pathway  Genes encoding collagen proteins  Cytokine-cytokine receptor interaction  Cytokine-cytokine receptor interaction  Cytokine-cytokine receptor interaction  Free Radical Induced Apoptosis  Cytokines can induce activation of matrix metalloproteinases which degrade extracellular matrix  Cytokines can induce activation of matrix metalloproteinases which degrade extracellular matrix  IL27-mediated signaling events  Cytokine-cytokine receptor interaction  Ensemble of genes encoding core extracellular matrix including ECM glycoproteins collagens and proteoglycans  VEGF Hypoxia and Angiogenesis  HIF-Fluid shear stress and atherosclerosis alpha transcription factor network  Cells and Molecules involved in local acute inflammatory response  VEGF Hypoxia and Angiogenesis  HIF-Fluid shear stress and atherosclerosis alpha transcription factor network  ATF-Fluid shear stress and atherosclerosis transcription factor network  Cytokine-cytokine receptor interaction  HIF-1 signaling pathway  IL23-mediated signaling events  IL23-mediated signaling events  amb2 Integrin signaling  Mechanism of Gene Regulation by Peroxisome Proliferators via PPARa(alpha)  The IGF-1 Receptor and Longevity  Cytokines can induce activation of matrix metalloproteinases which degrade extracellular matrix  IL27-mediated signaling events  Antifolate resistance  Fluid shear stress and atherosclerosis  Fluid shear stress and atherosclerosis  IL-17 signaling pathway  Free Radical Induced Apoptosis  Adhesion and Diapedesis of Granulocytes  Cytokines can induce activation of matrix metalloproteinases which degrade extracellular matrix  Toll-like receptor signaling pathway  Toll-like receptor signaling pathway  Th17 cell differentiation  PI3K-Akt signaling pathway  Cytokine-cytokine receptor interaction  Cytokine-cytokine receptor interaction  HIF-1 signaling pathway  IL-17 signaling pathway  IL-17 signaling pathway  IL-17 signaling pathway  IL-17 signaling pathway  Ensemble of genes encoding ECM-associated proteins including ECM-affilaited proteins ECM regulators and secreted factors  Focal adhesion  HIF-1 signaling pathway  Fluid shear stress and atherosclerosis  Fluid shear stress and atherosclerosis  Fluid shear stress and atherosclerosis  Fluid shear stress and atherosclerosis  Fluid shear stress and atherosclerosis  Fluid shear stress and atherosclerosis  Fluid shear stress and atherosclerosis  PI3K-Akt signaling pathway  IL-17 signaling pathway  Cytokine-cytokine receptor interaction  Cytokine-cytokine receptor interaction  Cytokine-cytokine receptor interaction  Cytokine-cytokine receptor interaction  Plasma membrane estrogen receptor signaling  Cells and Molecules involved in local acute inflammatory response  Fat digestion and absorption  lntegrins in angiogenesis  lntegrins in angiogenesis  amb2 Integrin signaling  Mechanism of Gene Regulation by Peroxisome Proliferators via PPARa(alpha)  Free Radical Induced Apoptosis  Adhesion and Diapedesis of Granulocytes  Ensemble of genes encoding ECM-associated proteins including ECM-affilaited proteins ECM regulators and secreted factors  Cells and Molecules involved in local acute inflammatory response  IL23-mediated signaling events  amb2 Integrin signaling  Cells and Molecules involved in local acute inflammatory response  HIF-1 signaling pathway  HIF-1 signaling pathway  Cytokine-cytokine receptor interaction  Cytokine-cytokine receptor interaction  Plasma membrane estrogen receptor signaling  IL23-mediated signaling events  IL23-mediated signaling events  Mechanism of Gene Regulation by Peroxisome Proliferators via PPARa(alpha)  Fluid shear stress and atherosclerosis  PI3K-Akt signaling pathway  IL23-mediated signaling events  IL23-mediated signaling events  Toll-like receptor signaling pathway  Cytokine-cytokine receptor interaction  Fluid shear stress and atherosclerosis  Cells and Molecules involved in local acute inflammatory response  IL-17 signaling pathway  IL-17 signaling pathway  Ensemble of genes encoding ECM-associated proteins including ECM-affilaited proteins ECM regulators and secreted factors  Fluid shear stress and atherosclerosis  HIF-1 signaling pathway  Phagosome  Phagosome  HIF-1 signaling pathway  HIF-1 signaling pathway  HIF-1 signaling pathway  Ensemble of genes encoding core extracellular matrix including ECM glycoproteins collagens and proteoglycans  Plasma membrane estrogen receptor signaling  Genes encoding enzymes and their regulators involved in the remodeling of the extracellular matrix  Ensemble of genes encoding core extracellular matrix including ECM glycoproteins collagens and proteoglycans  Fluid shear stress and atherosclerosis  Fluid shear stress and atherosclerosis  Fluid shear stress and atherosclerosis  Cytokine-cytokine receptor interaction  Plasma membrane estrogen receptor signaling  IL-17 signaling pathway  Cytokine-cytokine receptor interaction  Cytokine-cytokine receptor interaction  Cytokine-cytokine receptor interaction  Plasma membrane estrogen receptor signaling  Phagosome  Fluid shear stress and atherosclerosis  Fluid shear stress and atherosclerosis  Cytokine-cytokine receptor interaction  PI3K-Akt signaling pathway  Toll-like receptor signaling pathway  PI3K-Akt signaling pathway  Fluid shear stress and atherosclerosis  Fluid shear stress and atherosclerosis  HIF-1 signaling pathway  Ensemble of genes encoding ECM-associated proteins including ECM-affilaited proteins ECM regulators and secreted factors  PI3K-Akt signaling pathway  PI3K-Akt signaling pathway  HIF-1 signaling pathway  Complement and coagulation cascades  HIF-1 signaling pathway  Fluid shear stress and atherosclerosis  Complement and coagulation cascades | | Adhesion and Diapedesis of Granulocytes  Focal adhesion  Ensemble of genes encoding ECM-associated proteins including ECM-affilaited proteins ECM regulators and secreted factors  IL27-mediated signaling events  Focal adhesion  IL27-mediated signaling events  lntegrins in angiogenesis  HIF-Fluid shear stress and atherosclerosis-alpha transcription factor network  Genes encoding collagen proteins  IL-17 signaling pathway  VEGF Hypoxia and Angiogenesis  Protein digestion and absorption  IL-17 signaling pathway  IL27-mediated signaling events  Glypican 1 network  Adhesion and Diapedesis of Granulocytes  IL27-mediated signaling events  Antifolate resistance  Antifolate resistance  IL 5 Signaling Pathway  Protein digestion and absorption  Focal adhesion  Focal adhesion  Free Radical Induced Apoptosis  Glypican 1 network  Glypican 1 network  Signaling mediated by p38-alpha and p38-beta  IL23-mediated signaling events  VEGF Hypoxia and Angiogenesis  Cytokines can induce activation of matrix metalloproteinases which degrade extracellular matrix  Antifolate resistance  Adhesion and Diapedesis of Granulocytes  Signaling mediated by p38-alpha and p38-beta  Longevity regulating pathway  Hematopoietic cell lineage  Hematopoietic cell lineage  Hematopoietic cell lineage  VEGF Hypoxia and Angiogenesis  Angiopoietin receptor Tie2-mediated signaling  Ensemble of genes encoding ECM-associated proteins including ECM-affilaited proteins ECM regulators and secreted factors  Toll-like receptor signaling pathway  Toll-like receptor signaling pathway  Toll-like receptor signaling pathway  IL27-mediated signaling events  Antifolate resistance  IL27-mediated signaling events  HIF-Fluid shear stress and atherosclerosis-alpha transcription factor network  VEGF Hypoxia and Angiogenesis  HIF-Fluid shear stress and atherosclerosis-alpha transcription factor network  PI3K-Akt signaling pathway  Free Radical Induced Apoptosis  Adhesion and Diapedesis of Granulocytes  Cytokines can induce activation of matrix metalloproteinases which degrade extracellular matrix  Antifolate resistance  Angiopoietin receptor Tie2-mediated signaling  Glypican 1 network  Glypican 1 network  IL-17 signaling pathway  Cytokines can induce activation of matrix metalloproteinases which degrade extracellular matrix  IL27-mediated signaling events  Antifolate resistance  Plasma membrane estrogen receptor signaling  IL23-mediated signaling events  amb2 Integrin signaling  Glypican 1 network  Toll-like receptor signaling pathway  Free Radical Induced Apoptosis  Adhesion and Diapedesis of Granulocytes  Cytokines can induce activation of matrix metalloproteinases which degrade extracellular matrix  Antifolate resistance  Angiopoietin receptor Tie2-mediated signaling  amb2 Integrin signaling  ABC transporters  VEGF Hypoxia and Angiogenesis  HIF-Fluid shear stress and atherosclerosis-alpha transcription factor network  Angiopoietin receptor Tie2-mediated signaling  ATF-Fluid shear stress and atherosclerosis transcription factor network  Ensemble of genes encoding ECM-associated proteins including ECM-affilaited proteins ECM regulators and secreted factors  Ensemble of genes encoding ECM-associated proteins including ECM-affilaited proteins ECM regulators and secreted factors  IL27-mediated signaling events  Toll-like receptor signaling pathway  Ensemble of genes encoding ECM-associated proteins including ECM-affilaited proteins ECM regulators and secreted factors  Ensemble of genes encoding ECM-associated proteins including ECM-affilaited proteins ECM regulators and secreted factors  IL-17 signaling pathway  HIF-Fluid shear stress and atherosclerosis-alpha transcription factor network  Angiopoietin receptor Tie2-mediated signaling  Toll-like receptor signaling pathway  Th17 cell differentiation  amb2 Integrin signaling  Mechanism of Gene Regulation by Peroxisome Proliferators via PPARa(alpha)  Hematopoietic cell lineage  Hematopoietic cell lineage  HIF-Fluid shear stress and atherosclerosis-alpha transcription factor network  Angiopoietin receptor Tie2-mediated signaling  Toll-like receptor signaling pathway  Th17 cell differentiation  Hematopoietic cell lineage  Cells and Molecules involved in local acute inflammatory response  Cytokine-cytokine receptor interaction  Ensemble of genes encoding ECM-associated proteins including ECM-affilaited proteins ECM regulators and secreted factors  amb2 Integrin signaling  Hematopoietic cell lineage  ATF-Fluid shear stress and atherosclerosis transcription factor network  Ensemble of genes encoding ECM-associated proteins including ECM-affilaited proteins ECM regulators and secreted factors  Focal adhesion  Fat digestion and absorption  Hematopoietic cell lineage  Plasma membrane estrogen receptor signaling  lntegrins in angiogenesis  Mineral absorption  Focal adhesion  Genes encoding enzymes and their regulators involved in the remodeling of the extracellular matrix  amb2 Integrin signaling  lntegrins in angiogenesis  lntegrins in angiogenesis  Mineral absorption  Hematopoietic cell lineage  Focal adhesion  PI3K-Akt signaling pathway  Th17 cell differentiation  Ensemble of genes encoding ECM-associated proteins including ECM-affilaited proteins ECM regulators and secreted factors  lntegrins in angiogenesis  Hematopoietic cell lineage  Ensemble of genes encoding ECM-associated proteins including ECM-affilaited proteins ECM regulators and secreted factors  Toll-like receptor signaling pathway  Toll-like receptor signaling pathway  HIF-1 signaling pathway  PI3K-Akt signaling pathway  Toll-like receptor signaling pathway  Ensemble of genes encoding ECM-associated proteins including ECM-affilaited proteins ECM regulators and secreted factors  Ensemble of genes encoding core extracellular matrix including ECM glycoproteins collagens and proteoglycans  PI3K-Akt signaling pathway  Focal adhesion  Phagosome  Focal adhesion  Ensemble of genes encoding ECM-associated proteins including ECM-affilaited proteins ECM regulators and secreted factors  Phagosome  Cytokine-cytokine receptor interaction  Genes encoding enzymes and their regulators involved in the remodeling of the extracellular matrix  Ensemble of genes encoding ECM-associated proteins including ECM-affilaited proteins ECM regulators and secreted factors  Genes encoding enzymes and their regulators involved in the remodeling of the extracellular matrix  Ensemble of genes encoding ECM-associated proteins including ECM-affilaited proteins ECM regulators and secreted factors | 0.87500  0.81250  0.80556  0.80000  0.78571  0.71429  0.67857  0.67500  0.66667  0.65000  0.64286  0.62500  0.61607  0.60000  0.60000  0.58333  0.58333  0.58333  0.58333  0.56667  0.55385    0.54167  0.54167  0.53333  0.53333  0.53333  0.53333  0.52500  0.51136  0.50000  0.50000  0.50000  0.50000  0.50000  0.50000  0.50000  0.50000  0.48214  0.48214  0.48214    0.47619  0.47619  0.47619  0.47619  0.47619  0.47619  0.47500  0.46875  0.46875  0.46667  0.45833  0.45833  0.45833  0.45833  0.45395    0.44444  0.42424  0.41071  0.40476  0.40476  0.40476  0.40000  0.40000  0.40000  0.40000  0.40000  0.39583  0.39583  0.39583  0.39583  0.39286  0.39286  0.39286  0.39286  0.39286  0.39286  0.39286  0.38596  0.38596  0.38596  0.37500  0.37500  0.37500  0.36111  0.33333  0.33333  0.33333  0.33333  0.32500  0.32500  0.32500  0.32500  0.31667  0.31250  0.31111  0.31111  0.31111  0.30882  0.30100  0.30000  0.30000  0.30000  0.30000  0.28846  0.28750  0.28333  0.28333  0.27692  0.27692  0.27692  0.27574  0.27143  0.27143  0.26667  0.26250  0.26250  0.26250  0.26250  0.25882  0.25758  0.25595  0.25556  0.25556  0.24762  0.24359  0.22549  0.22500  0.22286  0.22222  0.21212  0.19022  0.17788  0.17763  0.16993  0.16667  0.15887  0.15873  0.14348  0.14091  0.13846  0.13636  0.12795 | |
